# Pontin arginine methylation by CARM1 is crucial for epigenetic regulation of autophagy

**DOI:** 10.1038/s41467-020-20080-9

**Published:** 2020-12-08

**Authors:** Young Suk Yu, Hijai R. Shin, Dongha Kim, Seon Ah Baek, Seon Ah Choi, Hyejin Ahn, Amen Shamim, Jeonghwan Kim, Ik Soo Kim, Kyeong Kyu Kim, Kyoung-Jae Won, Sung Hee Baek

**Affiliations:** 1 Creative Research Initiatives Center for Epigenetic Code and Diseases, Department of Biological Sciences, Seoul National University, Seoul, 08826 South Korea; 2 Department of Anatomy, College of Medicine, The Catholic University of Korea, Seoul, 06591 South Korea; 3 Department of Molecular Cell Biology, School of Medicine, Sungkyunkwan University, Suwon, 16419 South Korea; 4 Biotech Research and Innovation Centre (BRIC), University of Copenhagen, 2200 Copenhagen N, Denmark; 5 Novo Nordisk Foundation Center for Stem Cell Biology, DanStem, Faculty of Health and Medical Sciences, University of Copenhagen, 2200 Copenhagen N, Denmark; 6 Present Address: Department of Molecular and Cell Biology, University of California Berkeley, Berkeley, CA 94720 USA

**Keywords:** Biochemistry, Cell biology, Molecular biology

## Abstract

Autophagy is a catabolic process through which cytoplasmic components are degraded and recycled in response to various stresses including starvation. Recently, transcriptional and epigenetic regulations of autophagy have emerged as essential mechanisms for maintaining homeostasis. Here, we identify that coactivator-associated arginine methyltransferase 1 (CARM1) methylates Pontin chromatin-remodeling factor under glucose starvation, and methylated Pontin binds Forkhead Box O 3a (FOXO3a). Genome-wide analyses and biochemical studies reveal that methylated Pontin functions as a platform for recruiting Tip60 histone acetyltransferase with increased H4 acetylation and subsequent activation of autophagy genes regulated by FOXO3a. Surprisingly, CARM1-Pontin-FOXO3a signaling axis can work in the distal regions and activate autophagy genes through enhancer activation. Together, our findings provide a signaling axis of CARM1-Pontin-FOXO3a and further expand the role of CARM1 in nuclear regulation of autophagy.

## Abstract (web summary)

Epigenetic regulations of autophagy have emerged as mechanisms for maintaining cellular homeostasis. Here the authors reveal that the CARM1-Pontin-FOXO3a signaling axis can activate autophagy related genes through enhancer activation.

## Introduction

Autophagy is a conserved process that digests its cytoplasmic components through lysosomal degradation. This “self-eating” process is an essential mechanism for cell survival by eliminating protein aggregates and damaged organelles, as well as for maintaining nutrient homeostasis by replenishing insufficient nutrients^1,2^. Therefore, autophagy is essential for the protection of the organism against diverse pathologies, including cancer, neurodegeneration, and immune diseases^1,3–5^. Moreover, given that there is a strong connection between dysregulated autophagy and various diseases, an in-depth understanding of the regulatory mechanism of the process is highly needed.

Although autophagy is an event that occurs in the cytoplasm, accumulating evidence indicates that transcriptional and epigenetic regulations occurring in the nucleus also have important roles in autophagy^6,7^. While many transcription factors such as transcription factor EB (TFEB) and FOXO stimulate autophagy by regulating the expressions of various autophagy genes and lysosomal genes^8–10^, epigenetic regulations such as histone modifications help maintain the autophagic flux by changing chromatin status^11–14^.

One of the important epigenetic modifications involved in autophagy is arginine methylation. Arginine methylation is a post-translational modification regulating a wide range of cellular processes, including transcriptional regulation, signal transduction, RNA processing, DNA repair as well as autophagy^7,15^. Arginine methylation is catalyzed by the protein arginine methyltransferase (PRMT) family that transfers the methyl group from *S*-adenosylmethionine (SAM) to an arginine residue^15^. We have previously reported CARM1/PRMT4 functions as a critical coactivator of TFEB in the autophagic process^16^. Glucose starvation activates the AMPK–SKP2–CARM1 signaling cascade, which leads to the stabilization of CARM1 in the nucleus, followed by an increased H3R17me2 level. In response to starvation, CARM1 binds TFEB and functions as an essential coactivator for the activation of various autophagy genes and lysosomal genes^16^. However, the autophagy defects could not be explained by dysregulation of TFEB–CARM1 alone, and the increased arginine methylation of non-histone proteins in addition to histones was also observed under glucose starvation. This finding strongly suggests that CARM1 has additional partners for exerting its transcriptional and epigenetic functions in autophagy.

Pontin (also known as RUVBL1, TIP49A) is a chromatin-remodeling factor that possesses both ATPase and DNA helicase activities. Accumulating evidence support that Pontin functions as a coactivator for various transcription factors^17–20^. Post-translational modifications of Pontin are needed for exerting its transcriptional activities. For example, SUMOylated Pontin has been shown to function as a transcriptional coactivator of androgen receptor-mediated transcription in prostate cancer^21^. Also, the lysine methylation of Pontin increases the transcriptional activity of the hypoxia-inducible factor 1α (HIF-1α) in breast cancer^22^. Compared to Pontin functioning as a transcriptional coactivator, Reptin chromatin-remodeling factor functions as a transcriptional corepressor in various signaling pathways^23,24^.

Here, we identify Pontin as a substrate of CARM1 under glucose starvation. Arginine methylated Pontin at R333 and R339 residues specifically interacts with FOXO3a transcription factor for transcriptional activation of autophagy genes and maintains autophagic flux under glucose starvation. We have found important binding residues of FOXO3a for the recognition of arginine methylated Pontin that are essential for Pontin-FOXO binding. Finally, we show that the CARM1–Pontin–FOXO3a signaling axis activates autophagy genes along with Tip60 coactivator by increasing H4 acetylation.

## Results

### Pontin is arginine methylated by CARM1 at R333 and R339 residues

**Fig. 1:**
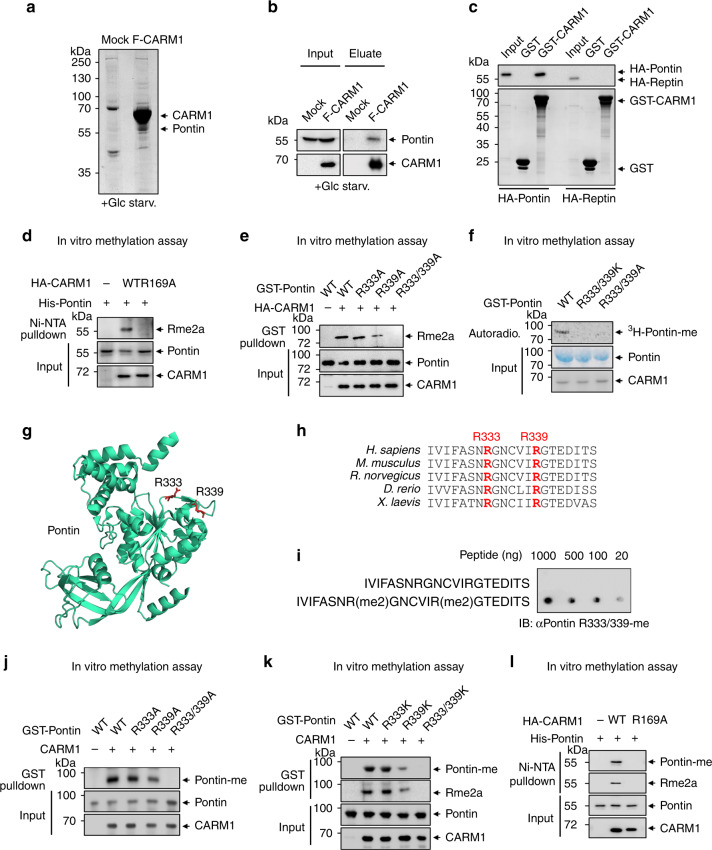
Pontin is arginine methylated by CARM1 at R333 and R339 residues. **a** CARM1-interacting proteins were purified from HEK293T cells stably expressing Flag-CARM1 by co-immunoprecipitation with Flag-M2 agarose. Mass spectrometric analysis identified Pontin as a CARM1-interacting protein under glucose starvation. **b** The binding of Pontin to CARM1 was detected from the eluate of affinity chromatography by immunoblot analysis. **c** GST-pulldown assay for binding of CARM1 to Pontin and Reptin. **d** In vitro methylation assay using purified His-Pontin was performed with either HA-CARM1 WT or enzymatic activity-deficient R169A mutant. Immunoblotting with anti-asymmetric dimethyl arginine (Rme2a) antibody was performed after Ni-NTA pulldown. **e** In vitro methylation assay using purified GST-Pontin WT, R333A, R339A, or R333/339A mutant with HA-CARM1. Immunoblot analysis with anti-Rme2a antibody was performed after GST pulldown. **f** In vitro methylation assay of Pontin WT, R333K/R339K mutant, or R333A/R339A mutant proteins was performed with CARM1 proteins. **g** Ribbon diagram of Pontin (PDB 2C9O) showing its structure with R333 and R339 residues. **h** Sequence alignment of human Pontin with selected homologs. Conserved arginine residues (R333/R339) are highlighted with red. **i** The specificity of antibodies against methylated Pontin at R333/R339 residues was assessed by dot blot analysis. Methyl peptide sequence: IVIFASNR(me2)GNCVIR(me2)GTEDITS; Non-methyl peptide sequence: IVIFASNRGNCVIRGTEDITS. **j** In vitro methylation assay using purified GST-Pontin WT, R333A, R339A, or R333/339A with CARM1. Immunoblot analysis with anti-methyl-Pontin antibody was performed after GST pulldown. **k** In vitro methylation assay using purified GST-Pontin WT, R333K, R339K, or R333/339K proteins with CARM1. Immunoblot analysis with anti-methyl-Pontin and anti-Rme2a antibody was performed following GST pulldown. **l** In vitro methylation assay using purified His-Pontin with HA-CARM1 WT or R169A. Immunoblot analysis with anti-methyl-Pontin antibody was performed after Ni-NTA pulldown. Source data are provided as a Source Data file.

To identify specific binding partners of CARM1 that could participate in the transcriptional regulation of autophagy during glucose starvation, we isolated CARM1-interacting proteins following glucose starvation by tandem affinity purification. Intriguingly, tandem mass spectrometry (LC–MS/MS) identified the Pontin chromatin-remodeling factor as a candidate for CARM1-interacting protein (Fig. 1a and Supplementary Table 1). The association of Pontin with CARM1 was further confirmed by immunoblot analysis from eluates using specific antibodies (Fig. 1b). To determine whether Pontin directly interacts with CARM1, we purified glutathione *S*-transferase (GST)-tagged CARM1 proteins and performed GST-pulldown assay with in vitro translated Pontin. Since Reptin has been shown to be a well-known binding partner of Pontin, we examined whether CARM1 can bind Reptin as well. GST-pulldown experiment revealed the selective direct binding of CARM1 to Pontin, but not to Reptin (Fig. 1c).

We conducted an in vitro methylation assay using purified proteins of CARM1 wild-type (WT) or enzymatically inactive R169A mutant to check the possibility that Pontin is arginine methylated by CARM1. Only CARM1 WT, but not R169A mutant, was able to methylate Pontin in vitro (Fig. 1d), indicating that enzymatic activity of CARM1 is important for exerting Pontin arginine methylation. To find methylation sites of Pontin by CARM1, we used programs for the prediction of methylation motifs^25^ and then replaced predicted arginines with alanines, either individually or in combination. While R333A or R339A mutant of Pontin showed reduced methylation, R333A/R339A double mutation almost completely diminished Pontin methylation (Fig. 1e). In parallel, we performed in vitro methylation assays using ^3^H-*S*-adenosylmethionine (SAM) with Pontin WT, R333K/R339K mutant, and R333A/R339A mutant and confirmed that Pontin WT, but not Pontin mutants, is directly methylated by CARM1 in vitro (Fig. 1f). Ribbon diagram of Pontin showed its structure with these two close R333 and R339 residues (Fig. 1g). Sequence alignment of Pontin among species revealed two arginine residues and surrounding amino acid sequences are highly conserved among species (Fig. 1h).

For further analysis, we generated an arginine methyl-Pontin antibody (anti-Pontin-me) that specifically recognizes the methylation at R333 and R339 residues. Dot blot analysis of methyl-Pontin antibody (Fig. 1i) and in vitro methylation assay with Pontin WT, R333A, R339A, and R333A/R339A double mutant (Fig. 1j) confirmed the specificity of the methyl-Pontin antibody. As in the case of R333A/R339A mutant, Pontin methylation was almost completely abolished by the introduction of R333K/R339K mutant (Fig. 1k). Further, in vitro methylation assay revealed that CARM1 WT, but not enzymatically inactive R169A mutant, was capable of Pontin methylation (Fig. 1l). Since there have been reports that Pontin is methylated by other arginine methyltransferase^26^, we checked whether the methyl-Pontin antibody captures CARM1-specific methylation. We observed that Pontin can bind PRMT5 and PRMT6 as well as CARM1/PRMT4 (Supplementary Fig. 1a), but the antibody recognizes methyl-Pontin only in CARM1-expressing, but not in other PRMT-expressing cells (Supplementary Fig. 1b). Together, these data indicate that CARM1 methylates Pontin at R333 and R339 residues both in vitro and in vivo.

### Pontin is arginine methylated by CARM1 in the nucleus upon glucose starvation

**Fig. 2:**
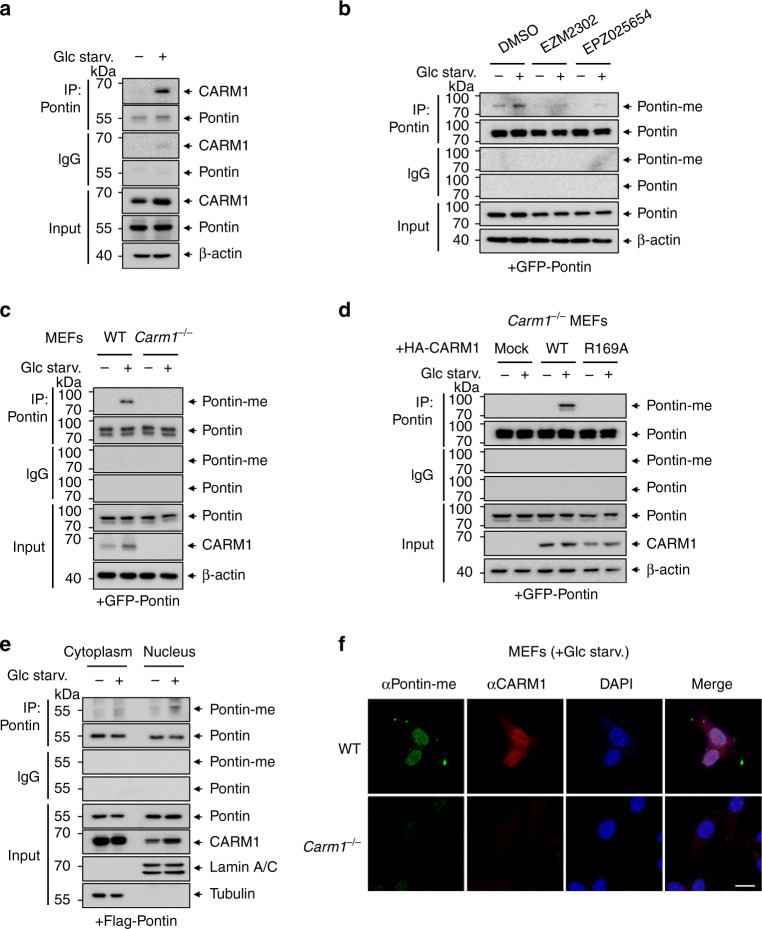
Pontin is arginine methylated by CARM1 in the nucleus upon glucose starvation. **a** Co-immunoprecipitation assay with Pontin and CARM1 in HeLa cells under glucose starvation for 18 h. **b** Pontin methylation in MEFs under glucose starvation was analyzed after treatment of CARM1-specific inhibitors, EZM2302 (100 nM) and EPZ025654 (100 nM) for 96 h. **c** Pontin methylation was evaluated in WT or *Carm1*^−/−^ MEFs following glucose starvation. **d** Pontin methylation was assessed in *Carm1*^−/−^ MEFs reconstituted with CARM1 WT or R169A mutant. **e** Pontin methylation was assessed in MEFs after nuclear and cytoplasmic fractionation. **f** Representative confocal images of methylated Pontin and CARM1. Scale bar, 20 μm. Source data are provided as a Source Data file.

As the protein levels of CARM1 increase following glucose starvation^16^, we checked whether Pontin–CARM1 interaction increases in response to starvation. As expected, endogenous Pontin showed increased binding to CARM1 following glucose starvation (Fig. 2a). We then sought to define whether increased interaction between Pontin and CARM1 during glucose starvation results in increased Pontin methylation. We observed that Pontin methylation increased in response to glucose starvation, whereas the methylation markedly decreased when treated with CARM1-specific inhibitors including EZM2302 and EPZ025654 (Fig. 2b). CARM1-dependent Pontin methylation increased in response to glucose starvation in WT mouse embryonic fibroblasts (MEFs) but not in *Carm1* knockout (KO) MEFs (Fig. 2c and Supplementary Fig. 1c). Further, Pontin methylation was restored in *Carm1* KO MEFs reconstituted with CARM1 WT, but not with CARM1 R169A mutant (Fig. 2d and Supplementary Fig. 1d), indicating that starvation-induced Pontin methylation is mediated by CARM1. In accordance with the previous report that starvation-induced CARM1 accumulation occurs in the nucleus^16^, cellular fractionation assay (Fig. 2e and Supplementary Fig. 1e) and immunocytochemistry analysis (Fig. 2f) confirmed that Pontin methylation by CARM1 occurs mainly in the nucleus in response to glucose starvation.

Because Pontin is known to form a hetero-dodecameric complex with its binding partner Reptin, we checked whether Pontin methylation affects the formation of the Pontin and Reptin complex. We performed an in vitro binding assay to examine whether methylation of Pontin at R333/R339 residues affects the formation of the Pontin–Reptin complex. Following in vitro methylation assay with Pontin, GST-pulldown assay was conducted to determine whether the methylation of Pontin affects the binding of Pontin to Reptin. GST-pulldown assay revealed that Pontin and Reptin were bound regardless of the methylation status of Pontin (Supplementary Fig. 1f). In addition, the binding to Reptin was not changed in the Pontin RK mutant (Supplementary Fig. 1g). Taken together, our data indicate that the methylation of Pontin does not alter the binding affinity to Reptin.

### Methylated Pontin is crucial for proper starvation-induced autophagy

**Fig. 3:**
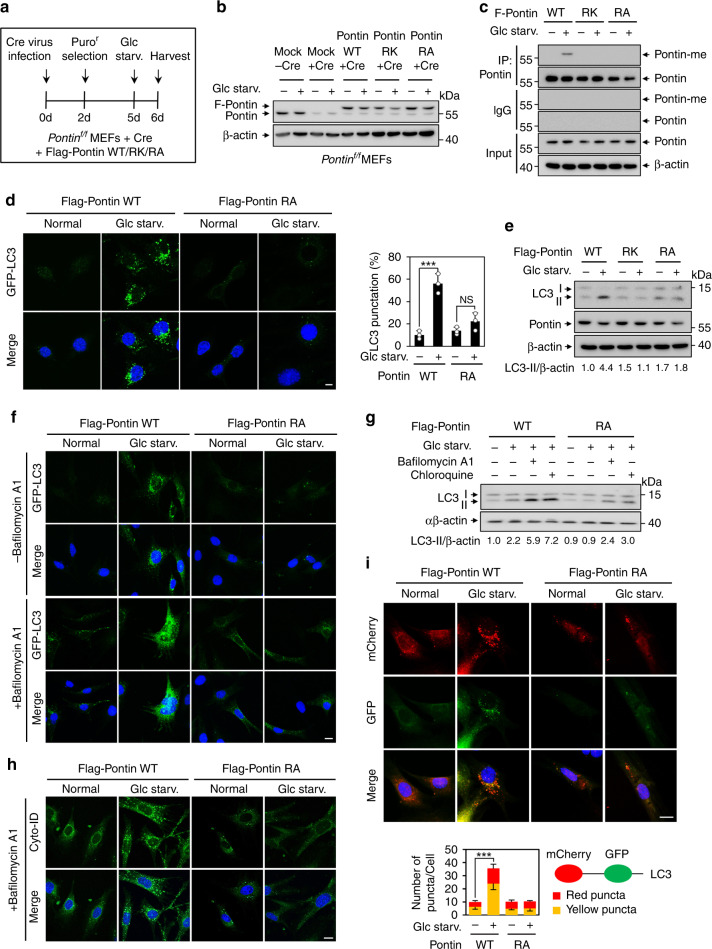
Methylated Pontin is crucial for proper starvation-induced autophagy. **a** Schematics for the generation of stably expressing Flag-Pontin WT, R333K/R339K (RK), or R333A/R339A (RA) mutant in *Pontin*^*f/f*^ MEFs. **b** The protein levels of Pontin were assessed by immunoblot analysis. **c** Immunoprecipitation assay against anti-Pontin-me antibody was performed following glucose starvation in *Pontin*^*f/f*^ MEFs stably expressing Flag-Pontin WT, RK, or RA mutant infected with Cre virus. **d** Representative confocal images of GFP-LC3 puncta formation. Scale bar, 20 μm. The graph shows the quantification of LC3-positive punctate cells (right). Bars, mean ± s.e.m.; *n* = 3 with over 150 cells; one-way ANOVA with post hoc Tukey’s test. ***p < 0.001. **e** Immunoblot analysis of LC3 levels in cell lysates of Pontin WT, RK, or RA MEFs upon glucose starvation. The number below indicates LC3-II/β-actin ratio. **f** Representative confocal images of GFP-LC3 puncta formation in the presence or absence of Bafilomycin A1 (200 nM; 2 h). Scale bar, 20 μm. **g** Autophagic flux was analyzed in Pontin WT or RA MEFs in the presence or absence of Bafilomycin A1 (200 nM; 2 h) or Chloroquine (10 μM; 4 h) under glucose starvation. The LC3-II/β-actin ratio is indicated. **h** Representative confocal images of autophagic vacuoles in Pontin WT or RA MEFS in the presence of Bafilomycin A1 (200 nM; 2 h). Autophagic vacuoles were detected using the CYTO-ID autophagy detection kit. Scale bar, 20 μm. **i** Representative confocal images of mCherry-GFP–LC3 assays in Pontin WT or RA MEFs. Colocalization between mCherry and GFP signal (yellow puncta) denotes autophagosomal vesicles that have not fused with a lysosomal compartment (phagophores or autophagosomes). mCherry signal without GFP signal (red puncta) denotes acidic autophagosomal vesicles (acidic amphisomes or autolysosomes). Scale bar, 20 μm. The graph indicates the number of yellow and red puncta per cell (right). Bars, mean ± s.e.m.; *n* = 10 biologically independent cells; ***p < 0.001. Statistics by two-tailed *t*-test. Source data are provided as a Source Data file.

To address the role of Pontin methylation during glucose starvation, we generated *Pontin*^*f/f*^ MEFs stably expressing Flag-tagged Pontin WT, R333K/R339K (RK), or R333A/R339A (RA) mutant (Fig. 3a). Cre virus infection results in the deletion of endogenous Pontin while leaving the ectopic Pontin unaffected, and the expression levels of stably expressing Flag-tagged Pontin WT, RK, or RA were similar to the endogenous level of Pontin (Fig. 3b). These cell lines were used throughout this study to assess the role of methylated Pontin in vivo. Pontin methylation was observed only in Pontin WT-expressing MEFs, but neither in Pontin RK mutant- nor in RA mutant-expressing MEFs (Fig. 3c). In addition, Pontin methylation increased by rapamycin treatment or amino acid starvation (Supplementary Fig. 2a, b). Considering the prominent role of CARM1 in autophagy, we sought to examine whether methylated Pontin is required for starvation-induced autophagic occurrence. We had previously observed that the GFP-LC3 punctate cells significantly decreased in the *Pontin* KO MEFs (Supplementary Fig. 2c). In order to observe autophagic occurrence according to the methylation status of Pontin, we performed the GFP-LC3 puncta assay using *Pontin* KO MEFs stably expressing Pontin WT or RA mutant. The increase in GFP-LC3 punctate cells upon glucose starvation was strikingly attenuated in Pontin RA MEFs compared to Pontin WT MEFs (Fig. 3d). The same results were observed upon rapamycin treatment or amino acid starvation (Supplementary Fig. 2d, e). Moreover, an increased ratio of the levels of lipidated LC3-II form to the levels of β-actin, a commonly used biological marker of autophagy, was observed in Pontin WT MEFs following glucose starvation, but neither in Pontin RK nor in Pontin RA MEFs (Fig. 3e).

Next, we performed autophagic flux analysis using Bafilomycin A1 or Chloroquine, two well-established inhibitors of the late phase of autophagy that prevent the maturation of autophagic vacuoles by inhibiting fusion between autophagosomes and lysosomes. GFP-LC3 puncta formation assay showed that the marked accumulation of GFP-LC3 puncta upon glucose starvation, when treated with Bafilomycin A1, was attenuated in Pontin RA MEFs compared to Pontin WT MEFs (Fig. 3f). Immunoblot analysis of LC3 confirmed that LC3-II was accumulated in Pontin WT MEFs but not in Pontin RA MEFs when autophagic flux was blocked by Bafilomycin A1 or Chloroquine treatment under glucose starvation (Fig. 3g), rapamycin treatment, and amino acid starvation (Supplementary Fig. 2f, g). Further, we used Cyto-ID dye, which specifically labels autophagic compartments. The autophagic vacuole staining using Cyto-ID dye showed similar results (Fig. 3h). The mCherry-GFP-LC3 reporter was used to assess the overall number of LC3 puncta formation as well as autophagic flux. Because the GFP signal is attenuated in the acidic lysosomal environment, while the mCherry is not, this reporter allows distinction between autophagosomal vesicles that have not fused with a lysosomal compartment (e.g., phagophores or autophagosomes) (GFP+/mCherry+ yellow puncta) and acidic autophagosomal vesicles (e.g., acidic amphisomes or autolysosomes) (GFP−/mCherry+ red puncta). Induction of the total numbers of both red and yellow puncta under glucose starvation was significantly attenuated in Pontin RA MEFs in contrast to Pontin WT MEFs (Fig. 3i). These data indicate that CARM1-mediated Pontin methylation is required for proper starvation-induced autophagy.

To explore whether Pontin methylation-dependent regulation of autophagy is a common regulatory mechanism of autophagy, we investigated if this regulation also works in other cell lines such as HepG2 and HeLa. Under glucose starvation, both cell lines showed an increase in Pontin methylation, including an increase in CARM1 in the nucleus (Supplementary Fig. 3a, b). To address the role of Pontin methylation in HepG2 and HeLa cell lines, we generated Pontin knockdown cells ectopically expressing shRNA-resistant Flag-tagged Pontin WT (WT^R^) or RA (RA^R^) mutant. We observed Bafilomycin A1-dependent accumulation of LC3-II upon glucose starvation in Pontin WT cells, but not in Pontin RA cells (Supplementary Fig. 3c, d). Our data indicate that autophagy regulation by Pontin methylation is a common mechanism of regulating autophagy in various cell lines.

Maintaining homeostasis of nutrient metabolism is critical for cells to be healthy and function normally. Autophagy is one of the important cellular defense mechanisms against nutrient deprivation and helps survival by providing nutrients to cells through self-consumption. To observe cell survival according to the methylation of Pontin, we analyzed the proliferation rate of Pontin WT or RK MEFs under glucose starvation. In the case of Pontin WT MEFs, we observed that the increase in proliferation no longer occurred around 15–18 h and the cells died rapidly after 24 h of glucose starvation. On the other hand, in the case of Pontin RK MEFs, there was no difference with Pontin WT MEFs in proliferation until 12 h, but cells died rapidly after 12 h of glucose starvation (Supplementary Fig. 3e). Next, we performed cell viability assays using Pontin WT or RK MEFs under glucose starvation. Consistent with proliferation assay, we observed cell viability meaningfully decreased at 24 h under glucose starvation in Pontin WT MEFs. On the other hand, in the case of Pontin RK MEFs, cell viability was significantly reduced after 18 h of glucose starvation (Supplementary Fig. 3f). Taken together, these data showing the difference in cell growth and viability based on Pontin methylation status further demonstrate the importance of transcriptional regulation of autophagy as a critical event for cell survival during prolonged starvation.

### RNA-sequencing analysis reveals that methylated Pontin-dependent genes are enriched for autophagy and lysosomal genes

**Fig. 4:**
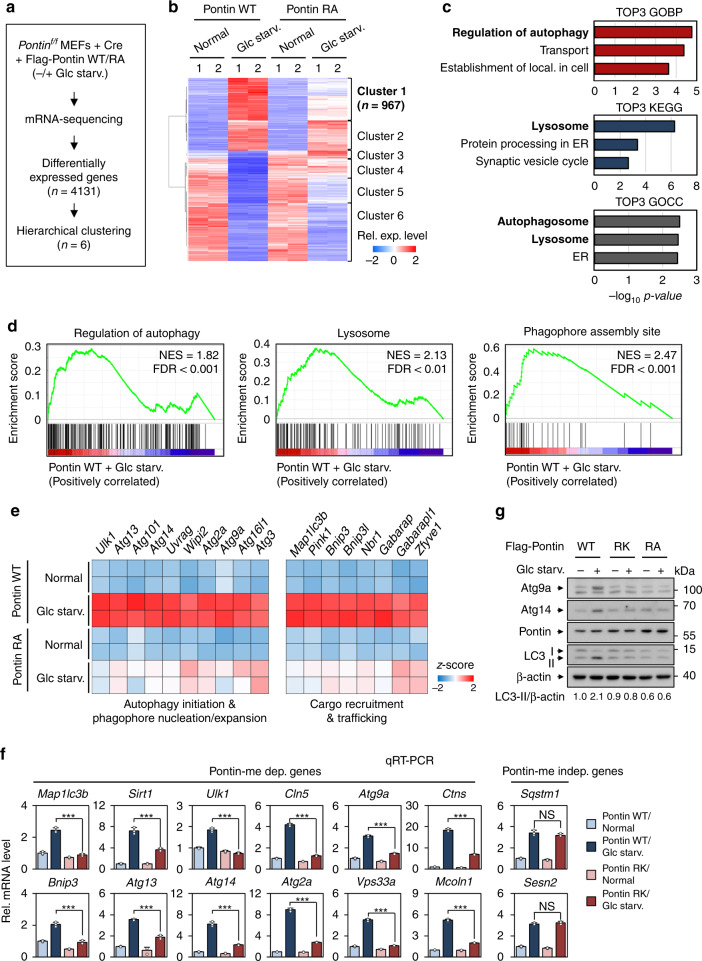
RNA-sequencing analysis reveals that methylated Pontin-dependent genes are enriched for autophagy and lysosomal genes. **a** Flow chart showing the strategy of RNA-sequencing analysis. **b** Hierarchical clustering results applied to 4131 differentially expressed genes (DEGs). **c** Gene ontology enrichment and KEGG pathways enrichment analyses for the genes in Cluster 1 using DAVID software. **d** Representative gene set enrichment analysis (GSEA) results (ranking genes by Pearson correlation with the phenotypic labels based on the Cluster 1). **e** Pontin methylation-dependent genes sorted by autophagy steps. Heatmaps representing expression changes of genes. **f** Quantitative RT-PCR analysis of methylated Pontin-dependent autophagy and lysosomal genes. Bars, mean ± s.e.m.; *n* = 3; ***p < 0.001, NS non-significant. Statistics by two-tailed *t*-test. **g** Immunoblot analysis of Pontin methylation-dependent genes in cell lysates of Pontin WT, RK, or RA MEFs upon glucose starvation. Source data are provided as a Source Data file.

Since Pontin methylation is markedly induced in the nucleus upon glucose starvation concomitant with CARM1 nuclear accumulation, we hypothesized that methylated Pontin is involved in transcriptional regulation of autophagy. To determine which genes are affected by methylated Pontin, we performed RNA-sequencing (RNA-seq) in Pontin WT and RA MEFs after glucose starvation (Fig. 4a). Principal component analysis (PCA) revealed that the global gene expression patterns between Pontin WT and RA MEFs are similar in normal conditions. However, glucose starvation significantly changed the global gene expression patterns between them (Supplementary Fig. 4a). We identified differentially expressed genes (DEGs) that can be classified into six clusters of gene groups by hierarchical clustering (Fig. 4b). Using enrichment analysis of gene ontology biological processes (GOBPs), gene ontology cellular component (GOCC), and Kyoto Encyclopedia of Genes and Genomes (KEGG) pathways, we found that the genes in Cluster 1 are significantly enriched for autophagy and lysosomal genes (Fig. 4c, Supplementary Fig. 4b, and Supplementary Table 2). In order to identify the subset of genes that actually contribute to the autophagy and lysosome, Gene Set Enrichment Analysis (GSEA) was performed through ranking genes by Pearson correlation with the phenotypic labels based on cluster 1 (Supplementary Fig. 5a). The results showed that the gene sets involved in the regulation of autophagy, lysosome, the phagophore assembly site are positively correlated with cluster 1 (Fig. 4d and Supplementary Fig. 5b).

Next, we explored how Pontin methylation has an important role in the autophagy process. RNA-sequencing data were systemically analyzed based on the genes that are important for each autophagy process. Interestingly, our analysis revealed that the expression of genes involved in autophagy initiation, phagophore nucleation/expansion, and cargo recruitment/trafficking was downregulated upon glucose starvation in Pontin RA MEFs in contrast to Pontin WT MEFs (Fig. 4e). Quantitative RT-PCR (qRT–PCR) and immunoblot analyses confirmed that the genes are regulated in Pontin methylation-dependent manner (Fig. 4f, g and Supplementary Fig. 6a). Consistent with the data in MEFs, other cell lines including HepG2 and HeLa showed similar results (Supplementary Fig. 6b, c). Under rapamycin treatment and amino acid starvation, the induction of autophagy and lysosomal genes was also observed in Pontin WT MEFs, but not in Pontin RA MEFs (Supplementary Fig. 6d, e). Together, our data indicate that autophagy and lysosomal genes are activated by Pontin methylation under starvation and that Pontin methylation has an important regulatory function in the autophagy process from the autophagy initiation step.

### Methylated Pontin binds FOXO3a through the arginine methyl-binding residues of FOXO3a

**Fig. 5:**
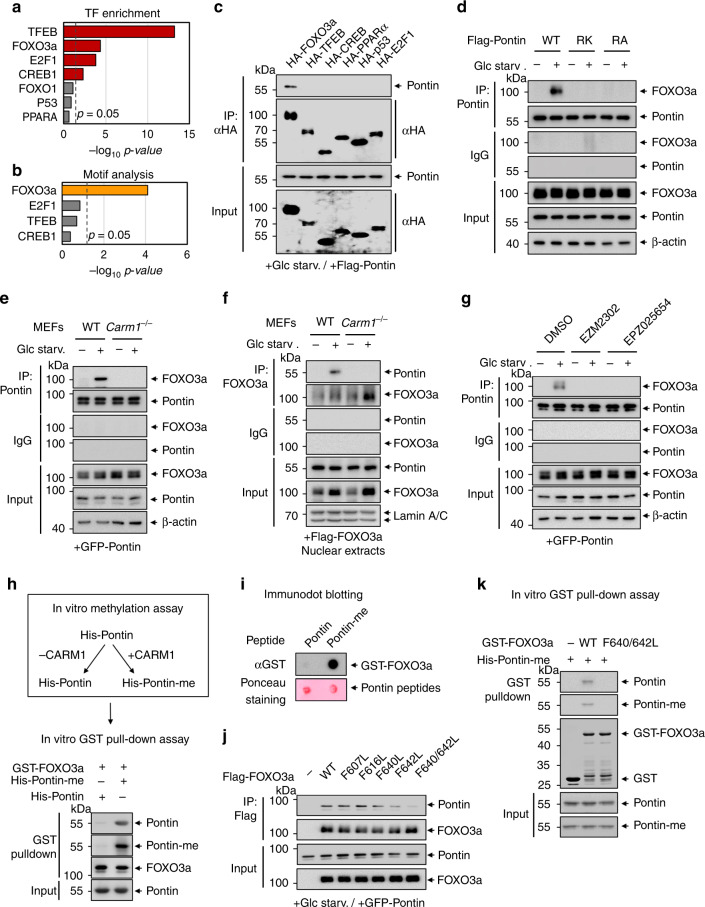
Methylated Pontin binds FOXO3a through the arginine methyl-binding domain of FOXO3a. **a** Transcription factor enrichment analysis for the methylated Pontin-dependent genes using EnrichR software. **b** Binding motif analysis using methylated Pontin ChIP-seq data. Hypergeometric *p*-values were calculated. **c** Co-immunoprecipitation assay was performed to detect the interaction between Pontin and various autophagy-related transcription factors upon glucose starvation. **d** Co-immunoprecipitation assay was performed to detect the interaction between FOXO3a and Pontin in Pontin WT, RK or RA-expressing MEFs. **e**, **f** Co-immunoprecipitation assay of FOXO3a with Pontin in WT or *Carm1*^−/−^ MEFs (**e**) or from nuclear extracts obtained from WT or *Carm1*^−/−^ MEFs (**f**) following glucose starvation. **g** Co-immunoprecipitation assay was performed to detect the interaction between FOXO3a and Pontin in MEFs under glucose starvation after treating with CARM1-specific inhibitors, EZM2302 (100 nM) and EPZ025654 (100 nM) for 96 h. **h** Scheme of in vitro methylation assay for preparing non-methylated and methylated Pontin (above). GST-pulldown assay was performed to examine the binding of FOXO3a to methylated Pontin or non-methylated Pontin (below). **i** Immunodot blot assay for the binding of GST-FOXO3a to Pontin or methylated Pontin peptide. **j** Co-immunoprecipitation assay of Pontin with FOXO3a WT or aromatic mutants following glucose starvation. **k** GST-pulldown assay for the binding of methylated Pontin to FOXO3a C terminus (418–673 aa) or F640/F642L mutant. Source data are provided as a Source Data file.

Since Pontin functions as a coactivator of various transcription factors, we examined whether Pontin, when arginine methylated, interacts with specific transcription factors. To find the major transcription factors for methylated Pontin-dependent genes obtained from RNA-seq data, transcription factor enrichment analysis was performed using web-based software, EnrichR^27^. The results of the transcription factor enrichment analysis helped us to narrow down the candidate transcription factors, which are known to be important for regulating autophagy processes upon glucose starvation (Fig. 5a). Furthermore, we performed chromatin immunoprecipitation with high-throughput sequencing (ChIP-seq) of methylated Pontin in WT MEFs after glucose starvation. When the motif analysis was performed through the DNA sequence recruited by methylated Pontin from the ChIP-seq data, the significance of the FOXO3a response elements was the highest (Fig. 5b). Individual binding assay further confirmed the selective binding of Pontin to FOXO3a, but not to TFEB, CREB, PPARα, p53, and E2F1, following glucose starvation (Fig. 5c). This interaction is Pontin methylation-dependent, as only Pontin WT bound FOXO3a, whereas Pontin RK and RA mutant failed to do so (Fig. 5d). To further examine whether Pontin methylation is required for the binding of Pontin to FOXO3, we checked whether CARM1 affects their binding. Interaction between Pontin and FOXO3a markedly increased in response to glucose starvation in WT MEFs but not in *Carm1* KO MEFs (Fig. 5e, f). Treatment with the CARM1-specific inhibitors markedly decreased the interaction of Pontin and FOXO3a (Fig. 5g), indicating that CARM1-dependent Pontin methylation is required for the binding to FOXO3a.

Next, we conducted an in vitro GST-pulldown assay to check whether methylated Pontin directly binds FOXO3a. After in vitro methylation assay with His-Pontin, we performed a GST-pulldown assay to determine whether the binding of Pontin to FOXO3a depends on the methylation status of Pontin. GST-pulldown assay revealed that methylated Pontin, but not non-methylated Pontin, directly binds FOXO3a (Fig. 5h). Further, we performed immunodot blot analysis using non-methylated or methylated Pontin peptides as bait and purified FOXO3a proteins as prey. FOXO3a selectively bound methylated Pontin peptide, but not non-methylated Pontin peptide, indicating that Pontin methylation is crucial for Pontin–FOXO3a binding (Fig. 5i). Further, we mapped the regions of FOXO3a necessary for its association with Pontin using a set of truncated FOXO3a proteins. The co-immunoprecipitation assay revealed that methylated Pontin specifically bound the C terminus of FOXO3a (418–673 a.a.) (Supplementary Fig. 7a). Further analysis showed that methylated Pontin specifically bound the CR3 domain of FOXO3a encompassing 610–650 amino acids upon glucose starvation (Supplementary Fig. 7b).

It has been shown that the methylated arginine can be recognized by the aromatic residues present in the arginine methyl reader domain. For example, TDRD3 contains a Tudor domain that specifically recognizes H3R17me2, H4R3me2, and arginine methylated RNA polymerase II CTD^28–30^. Both JMJD5 and JMJD7 contain aromatic residues and a negatively charged pocket that recognizes methylated arginine^31^. Therefore, we expected that FOXO3a possesses the aromatic residues within the CR3 domain that are responsible for recognizing the methylated arginine in Pontin. Therefore, we made all the aromatic residue mutants present in the CR3 domain to examine whether the FOXO3a mutants bind to Pontin. While F640L or F642L mutant of FOXO3a showed slightly reduced binding to Pontin, F640L/F642L double mutant showed almost abolished binding to Pontin (Fig. 5j). Furthermore, in vitro GST-pulldown assay confirmed that FOXO3a F640L/F642L double mutant failed to interact with methylated Pontin (Fig. 5k), indicating that two aromatic residues (F640 and F642) of FOXO3a are crucial for its interaction with methylated Pontin.

On the other hand, the modeling of methylated Pontin with FOXO3a predicted that FOXO3a possesses hydrophobic residues (M624, I627, and I628) that can function as a possible methyl-binding domain (Supplementary Fig. 7c). We, therefore, assessed the binding of arginine methylated Pontin to FOXO3a using FOXO3a WT or 3A (M624A, I627A, and I628A) mutant with compromised methyl-binding domain. To prove the direct binding between Pontin and FOXO3a, we performed in vitro GST-pulldown assays using FOXO3a WT or 3A mutant. GST-pulldown assays revealed that FOXO3a WT, but not 3A mutant, directly binds methylated Pontin (Supplementary Fig. 7d). Further, the co-immunoprecipitation assay confirmed that Pontin only bound FOXO3a WT but not FOXO3a 3A mutant (Supplementary Fig. 7e). Our data indicate that three residues (M624, I627, and I628) of FOXO3a are also crucial for interaction with methylated Pontin.

### Methylated Pontin and Tip60 activate FOXO3a target genes with histone H4 acetylation

**Fig. 6:**
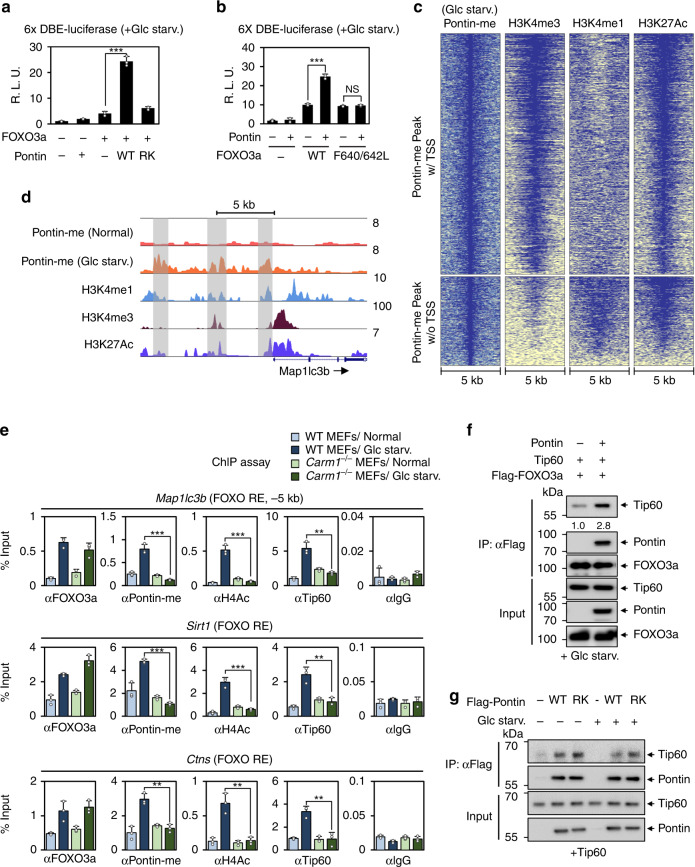
Methylated Pontin and Tip60 activate FOXO3a target genes with histone H4 acetylation. **a** Effect of Pontin WT or RK on 6x DBE (FOXO RE)-luciferase reporter. Bars, mean ± s.e.m; *n* = 3; ***p < 0.001. Statistics by two-tailed *t*-test. **b** Effect of FOXO3a WT, or F640L/F642L mutant on 6× DBE-luciferase reporter. Bars, mean ± s.e.m; *n* = 3; ***p < 0.001, NS non-significant. Statistics by one-way ANOVA with post hoc Tukey’s test. **c** Heatmap showing methylated Pontin near the TSS (above) or distally located to TSS (below). The data on H3K4me1, H3K4me3, and H3K27ac were obtained from MEFs under normal conditions. **d** Profiles of methylated Pontin (Normal), methylated Pontin (Glc starv.), H3K4me1, H3K4me3, and H3K27Ac ChIP-seq occupancy in the locus of the *Map1lc3b* gene. Gray boxes indicate possible Pontin binding sites. **e** ChIP assays were performed using anti-FOXO3a, anti-Pontin-me, anti-H4ac, anti-Tip60, and anti-IgG antibodies on FOXO REs in WT or *Carm1*^−/−^ MEFs in the absence or presence of glucose starvation. Bars, mean ± s.e.m.; *n* = 3; **p < 0.01, ***p < 0.001. Statistics by two-tailed *t*-test. **f** Co-immunoprecipitation assay between FOXO3a and Tip60 in the absence or presence of Pontin. **g** Co-immunoprecipitation assay between Tip60 and Pontin WT or RK mutants in the absence or presence of glucose starvation. Source data are provided as a Source Data file.

To examine whether methylated Pontin functions as a coactivator of FOXO3a, we tested the effect of methylated Pontin on luciferase reporter bearing FOXO-binding sites. The luciferase activity increased when Pontin is methylated upon glucose starvation, but it failed to do so in the presence of a Pontin RK or RA mutant (Fig. 6a and Supplementary Fig. 7f). In addition, the transcriptional activation function of Pontin was significantly attenuated by FOXO3a F640L/F642L and 3A mutants that are unable to bind Pontin (Fig. 6b and Supplementary Fig. 7g).

In order to find how methylated Pontin regulates FOXO3a target genes, the ChIP-seq peaks for methylated Pontin were examined. We observed that a number of methylated Pontin peaks were detected far from the TSS, where possessing the characteristics of the enhancers with increased H3K4me1 and H3K27Ac levels, as well as at the proximal region within 2.5 kb of TSS (Fig. 6c). Interestingly, of the methylated Pontin peaks with a FOXO3a motif, 36% of the peaks were far from the TSS. To observe how methylated Pontin is recruited, we checked the methylated Pontin peaks at FOXO3a target genes. We observed an increase of methylated Pontin levels in response to glucose starvation at not only the proximal region but also distal regions of the *Map1lc3b* gene locus (Fig. 6d). To examine whether FOXO3a and Pontin are recruited at the peaks observed above, we performed ChIP assays and found that recruitment of both FOXO3a and methylated Pontin increased at distal regions (−11.3 and −5 kb from TSS) as well as proximal region (−1.2 kb from TSS) of *Map1lc3b* upon glucose starvation (Supplementary Fig. 8a). However, in Pontin RA MEFs, while the recruitment of FOXO3a increased in response to glucose starvation, Pontin failed to be recruited on FOXO response elements (FOXO RE) (Supplementary Fig. 8a). These data indicate that methylated Pontin functions as a coactivator for FOXO3a, as Pontin acquired the binding ability to FOXO3a through arginine methylation.

Since transcription of autophagy genes was activated by the binding of methylated Pontin to FOXO3a, we explored the molecular mechanism of Pontin acting as a transcriptional coactivator of FOXO3a for autophagy genes. Pontin is one of the components of the Tip60 complex and is required for the acetyltransferase activity of Tip60 and subsequent transcriptional activation^21,26,32–34^. Therefore, we examined the possible recruitment of Tip60 and increased H4 acetylation on the FOXO RE containing genes. ChIP assays showed recruitment of Tip60 and increased histone H4 acetylation on methylated Pontin-dependent FOXO3a target genes in response to glucose starvation (Fig. 6e and Supplementary Fig. 8a, b). Interestingly, the recruitment of Tip60 with increased H4 acetylation was dependent on the methylation status of Pontin, as their recruitment was abolished in *Carm1* KO MEFs upon glucose starvation (Fig. 6e). The co-immunoprecipitation assay revealed that the binding of FOXO3a to Tip60 increased in the presence of Pontin (Fig. 6f), while Pontin binds to Tip60 regardless of its methylation status (Fig. 6g). Taken together, these data indicate that methylated Pontin and Tip60 function as coactivators of FOXO3a for autophagy genes and the binding of Tip60 to FOXO3a is Pontin methylation-dependent.

### Tip60 is crucial for the activation of methylated Pontin-dependent genes for autophagy

**Fig. 7:**
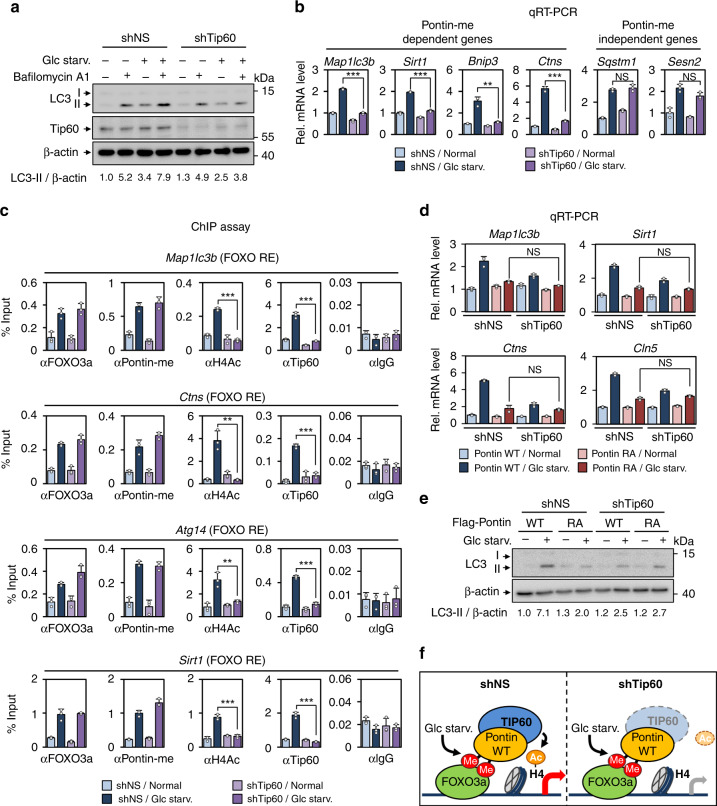
Pontin and Tip60 complex is important for autophagic occurrence through the regulation of FOXO3a target genes. **a** Autophagic flux was analyzed in MEFs after knocking down with shRNA for Tip60 (shTip60) or non-specific shRNA (shNS) in the presence or absence of Bafilomycin A1 (200 nM; 2 h) under glucose starvation. The LC3-II/β-actin ratio is indicated. **b** Quantitative RT-PCR analysis of methylated Pontin-dependent genes and independent genes in MEFs by knocking down with either shNS or shTip60 in the absence or presence of glucose starvation. Bars, mean ± s.e.m; *n* = 3; **p < 0.01, ***p < 0.001, NS non-significant. Statistics by two-tailed *t*-test. **c** ChIP assays were performed using anti-FOXO3a, anti-Pontin-me, anti-H4ac, anti-Tip60, and anti-IgG antibodies on FOXO REs in MEFs by knocking down with either shNS or shTip60 in the absence or presence of glucose starvation. Bars, mean ± s.e.m; *n* = 3; **p < 0.01, ***p < 0.001. Statistics by two-tailed *t*-test. **d** Quantitative RT-PCR analysis of methylated Pontin-dependent autophagy and lysosomal genes in Pontin WT or RA MEFs with or without Tip60 knockdown. Bars, mean ± s.e.m; *n* = 3; NS non-significant. One-way ANOVA with post hoc Tukey’s test. **e** Immunoblot analysis of LC3 levels in Pontin WT or RA MEFs with or without Tip60 knockdown. The LC3-II/β-actin ratio is indicated. **f** Schematics for the transcriptional activation of FOXO3a and methylated Pontin-dependent autophagy genes regulated by Tip60 with increased H4 acetylation upon glucose starvation. Glucose starvation-induced methylated Pontin by CARM1 at R333 and R339 residues binds FOXO3a through a methyl binding domain of FOXO3a along with Tip60 following increased H4 acetylation. Source data are provided as a Source Data file.

To determine whether Tip60 is crucial for the activation of methylated Pontin-dependent genes in autophagy, we knocked down Tip60 in MEFs using shRNA. Autophagic flux analysis using Bafilomycin A1 revealed that the accumulation of LC3-II upon glucose starvation was attenuated in Tip60 knockdown MEFs compared to control MEFs (Fig. 7a), indicating that Tip60 is involved in the regulation of starvation-induced autophagic occurrence. Next, we performed the qRT–PCR analysis of autophagy genes to examine whether knockdown of Tip60 leads to the decreased mRNA levels of methylated Pontin-dependent genes. Glucose starvation induced the mRNA levels of autophagy genes including *Map1lc3b, Sirt1*, *Bnip3*, and *Ctns*, but knockdown of Tip60 significantly attenuated the mRNA levels of these genes (Fig. 7b). Furthermore, we observed that autophagy genes that are methylated Pontin-independent were not affected by Tip60 knockdown (Fig. 7b). These data indicate that Tip60 and methylated Pontin collaborate for exerting transcriptional activation function of autophagy genes. Next, we performed ChIP assays on FOXO RE of these genes using anti-FOXO3a, Pontin-me, H4ac, and Tip60 antibodies along with control IgG to examine whether methylated Pontin and Tip60 are recruited to FOXO RE through FOXO3a and whether knockdown of Tip60 alters the recruitment of these factors. ChIP assays revealed that knockdown of Tip60 almost completely diminished increased H4 acetylation without affecting the recruitment of FOXO3a nor methylated Pontin (Fig. 7c).

Additionally, knockdown of Tip60 in Pontin WT MEFs showed a significant reduction of FOXO target gene activation following glucose starvation, but knockdown of Tip60 in Pontin RA MEFs did not show further downregulation of FOXO target genes, compared to Pontin RA MEFs infected with control shRNA (shNS) (Fig. 7d). These results further confirmed that Tip60-mediated H4 acetylation on FOXO RE is strictly mediated by Pontin methylation and that Tip60 exerts transcriptional activation function via Pontin methylation. In parallel, immunoblot analysis of LC3 revealed that the accumulation of LC3-II upon glucose starvation was significantly attenuated in Pontin RA MEFs or Tip60 knockdown MEFs (Fig. 7e). Taken together, the methylation status of Pontin is required for Tip60 recruitment on FOXO RE, and Tip60 recruitment and subsequent histone H4 acetylation are crucial for transcriptional activation of autophagy genes (Fig. 7f).

### CARM1–Pontin–FOXO3a signaling axis works for enhancer activation of autophagy and lysosomal genes

Previously, we demonstrated that AMPK mediated FOXO3a phosphorylation is critical for suppressing Skp2, the E3 ligase that degrades CARM1. To investigate the effect of Pontin on the Skp2 regulation, we performed quantitative RT-PCR (qRT–PCR) and ChIP assays. We observed a reduction of *Skp2* mRNA levels under glucose starvation regardless of the methylation of Pontin (Supplementary Fig. 9a). In addition, while FOXO3a was recruited on the *Skp2* promoter, the recruitment of Pontin along with H4 acetylation was not observed (Supplementary Fig. 9b). Considering that the increase of CARM1 is also observed in the methylated Pontin binding mutant of FOXO3a, it is thought that the methylation of Pontin has no effect on the increase of CARM1 including Skp2 (Supplementary Fig. 9c). Moreover, we have previously defined the role of CARM1 in autophagy as a coactivator of TFEB which binds the CLEAR motif of target promoters along with H3R17 dimethylation^16^. Knowing that both FOXO3a and TFEB are important for the transcription of the *Map1lc3b* gene, we tested the possibility that the CARM1–Pontin–FOXO3a signaling axis and the TFEB–CARM1 signaling axis work for combinatorial and cooperative activation of the same target genes but through each response element independently.

**Fig. 8:**
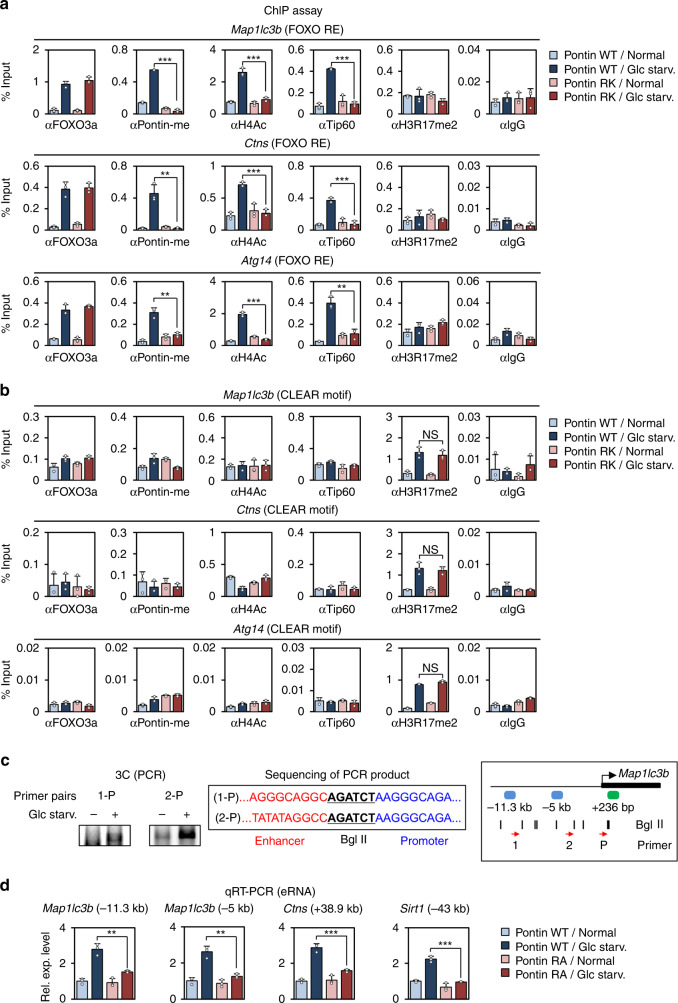
CARM1–Pontin–FOXO3a signaling axis works for enhancer activation of autophagy and lysosomal genes. **a**, **b** ChIP assays were performed using anti-FOXO3a, anti-Pontin-me, anti-H4ac, anti-Tip60, anti-H3R17me2, and anti-IgG antibodies on FOXO REs (**a**) or CLEAR motif (**b**) of *Map1lc3b*, *Ctns*, or *Atg14* genes in Pontin WT or RK-expressing MEFs in the absence or presence of glucose starvation. Bars, mean ± s.e.m; *n* = 3; **p < 0.01, ***p < 0.001, NS non-significant. Statistics by two-tailed *t*-test. **c** Chromosome conformation capture (3C) assay was performed to detect chromatin–chromatin interactions between the promoter and distal regions of *Map1lc3b* gene under glucose starvation. PCR products from the 3C assay were run on agarose gels. Sequence of the PCR product with Bgl II site is indicated (left). The model depicts *Map1lc3b* promoter with possible enhancer elements (blue blocks). Black linear lines represent Bgl II restriction sites, and red arrows represent primer used in 3C assay (right). **d** Quantitative RT-PCR analysis of methylated Pontin-dependent eRNAs for autophagy and lysosomal genes in the absence or presence of glucose starvation. Bars, mean ± s.e.m; *n* = 3; **p < 0.01, ***p < 0.001. Statistics by two-tailed *t*-test. Source data are provided as a Source Data file.

Since CARM1 is involved in both elements, we tested whether CARM1 is recruited to the FOXO3a REs and CLEAR motifs. ChIP assay revealed the increased CARM1 and H3R17me2 levels on the CLEAR motifs, but not on FOXO3a REs (Supplementary Fig. 10a, b). These data suggest that CARM1 is not directly recruited to the FOXO3a REs, although CARM1 is responsible for Pontin arginine methylation. Next, ChIP assays were performed to understand how FOXO3 REs and CLEAR motif are regulated by the methylation status of Pontin. Consistent with the previous results, FOXO3a was recruited on FOXO3 REs upon glucose starvation, and then methylated Pontin and Tip60 were co-recruited along with increased H4 acetylation (Fig. 8a). However, the introduction of Pontin methylation mutant almost completely blocked Tip60 recruitment along with H4 acetylation, as Pontin RK mutant failed to bind FOXO3a. Meanwhile, H3R17me2 did not change on the FOXO3a REs (Fig. 8a). On the other hand, on the CLEAR motif, FOXO3a, as well as methylated Pontin, were not recruited, while H3R17me2 level was significantly induced upon glucose starvation regardless of the methylation status of Pontin (Fig. 8b). Taken together, these data demonstrate that the CARM1–Pontin–FOXO3a signaling axis and the TFEB–CARM1 signaling axis work for activation of the same target genes but through each response element independently.

FOXO3a has been shown to bind enhancer regions and amplify enhancer activity for transcriptional activation of target genes^35^. Therefore, we hypothesized that the Pontin–FOXO3a signaling axis, which also regulates distal regulatory regions far from TSS, might regulate target genes through enhancer activation. To examine whether the enhancers function in a glucose starvation-dependent manner, we performed a chromosome conformation capture (3C) assay. Through the 3C assay, we found that the FOXO3a–Pontin binding regions of −11.3 kb and −5 kb from the transcription start site of *Map1lc3b* gene interact with the promoter in a glucose starvation-dependent manner (Fig. 8c). The PCR products were sequenced and confirmed to be the expected enhancer–promoter sequence with Bgl II restriction site. Next, in order to measure the activities of enhancers, expression of enhancer RNAs (eRNAs) was detected by qRT–PCR (Fig. 8d). Interestingly, the expression of eRNAs increased under glucose starvation in WT MEFs, but not in Pontin RA MEFs. Taken together, these data indicate that the CARM1–Pontin–FOXO3a signaling axis activates enhancer by increasing H4 acetylation.

## Discussion

**Fig. 9:**
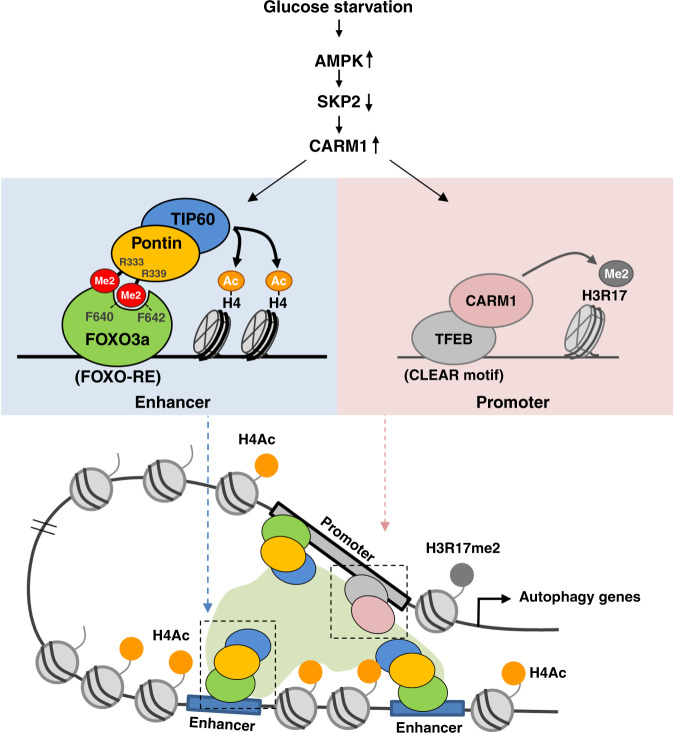
Schematic representation of CARM1–Pontin–FOXO3a signaling axis and CARM1–TFEB signaling axis for transcriptional activation. Schematics for the transcriptional activation of FOXO3a-dependent autophagy and lysosomal genes upon glucose starvation. Activated CARM1 methylates Pontin, and then methylated Pontin binds FOXO3a through a methyl-binding domain of FOXO3a with Tip60 and increased H4 acetylation. CARM1–Pontin–FOXO3a signaling axis works for FOXO3a response elements with increased H4 acetylation, whereas TFEB–CARM1 signaling axis works for CLEAR motifs with increased H3R17me2 level irrespective of Pontin methylation status.

Recent studies have unveiled the importance of epigenetic and transcriptional regulation in autophagy^6,7^. Continuous supply of the autophagy and lysosomal proteins, especially for sustained autophagy, is precisely controlled at the transcriptional level through a complex network of transcription factors and chromatin-remodeling factors in a combinatorial manner. The interplay among these factors needs to be tightly regulated for the proper autophagic occurrence and unraveling this complex network is important for understanding the process of autophagy. Our work here extends the roles of CARM1 in the epigenetic and transcriptional regulation of autophagy and reveals that Pontin, when arginine methylated by CARM1, functions as a positive regulator of autophagy. This methylated Pontin promotes interaction with FOXO3a, and recruitment of methylated Pontin to FOXO RE along with Tip60 and increased histone H4 acetylation leads to the induction of autophagy genes (Fig. 9).

Pontin is known to form a hetero-dodecameric complex with its binding partner Reptin. However, it has been known that Pontin and Reptin can independently control transcriptional regulation. One of the remaining questions is how Pontin and Reptin work independently and even act opposite each other. Our and other groups’ researches revealed that Pontin and Reptin are regulated differently through SUMOylation and lysine methylation. It has been reported that Pontin is methylated by PRMT5 at R205, whereas Reptin can be methylated by PRMT1^26^. We believe that the functional differences between Pontin and Reptin may be explained through various modifications including arginine methylation.

Although the current study focuses on the function of methylated Pontin in autophagy activation, it is highly possible that the interaction between FOXO3a and methylated Pontin is conserved in other physiological settings where FOXO3a is transcriptionally active and essential. It is also likely that these arginine methyl-binding residues are open to other methylated regulators of FOXO3a, allowing adequate and fine-tuned responses to different environmental stimuli. Interestingly, the arginine methyl-binding residues identified in this study is conserved among the FOXO transcription factors, suggesting that each FOXO family member could possess methyl-binding function. Since FOXOs have important roles under stress responses, finding new connections between FOXO family members and methylated partners will pave a new way for cells to cope with various stresses.

Nutrient deprivation, which restricts acetyl-CoA production from glucose or acetate uptake, leads to a marked reduction of global histone acetylation. Hypo-acetylation leads to the compaction of the chromatin structure, resulting in the suppression of gene expression. However, it has been reported that histone acetylation increases for genes important for survival such as autophagy genes, but the molecular mechanism remains unclear^36–38^. Given that FOXO3a–Pontin–Tip60 is recruited to autophagy genes and increases histone H4 acetylation to activate the expression of autophagy genes, it is tempting to speculate that the FOXO3–Pontin–Tip60 axis can be one of the answers to the histone acetylation rearrangement.

It is a classic view that enhancers bind promoters through transcriptional factors, mediators, and coactivators to activate transcription cooperatively^39^, but it is very difficult to define enhancers because of their remotely regulating characteristics. For this reason, how enhancers work to regulate autophagy genes in autophagic occurrence has not been studied thus far. In this study, we report that methylated Pontin can bind the distal DNA region as well as the promoter region via FOXO3a binding. By applying both 3C assay and qRT–PCR analysis of eRNAs, we found that in some autophagy genes, these distal regions actually act as enhancers. Although further investigation is still needed to understand how enhancers are orchestrated to work with promoters to regulate autophagy genes upon glucose starvation, methylated Pontin–Tip60 complex via FOXO3 may provide a prototype to the enhancer studies.

Numerous reports have proposed that combinatorial transcriptional activation of autophagy by TFEB and FOXO is important for life span extension^40^. Our current and previous studies propose CARM1 as an essential regulator of both transcription factors, either by directly functioning as a coactivator for TFEB with increased H3R17 methylation or by methylating Pontin, a coactivator of FOXO, with increased H4 acetylation by Tip60 coactivator. Consistent with decreased autophagy flux during aging, nuclear CARM1 has been shown to be downregulated in aged mice^41^. The future challenge would be to determine the impact of CARM1 in autophagy in relation to aging and age-related diseases such as cancer and neurodegenerative diseases. Further, pharmacological manipulation can provide beneficial and therapeutic autophagy activation by selectively inducing various transcription factors, coactivators, or chromatin-remodeling factors to activate various signaling axis.

## Methods

### Reagents

The following commercially available antibodies were used: anti-Pontin (SAB4200194), anti-Flag (F3165), and anti-β-actin (A1978) (Sigma-Aldrich); anti-Pontin (sc-15259), anti-GST (sc-459), anti-GFP (sc-9996), and anti-Lamin A/C (sc-6215) (Santa Cruz Biotechnology); anti-Tubulin (LF-PA0146A) (Abfrontier); anti-asymmetric dimethyl arginine motif (Rme2a) (#13522), anti-CARM1 (#3379), anti-LC3 (#2775), anti-FOXO3a (#2497), anti-phospho-FOXO3a S413 (#8174), and anti-Tip60 (#12058) (Cell Signaling Technology); anti-FOXO3a (ab12162) and anti-H3R17me2 (ab8284) (Abcam); anti-acetyl-Histone H4 (06-866) (Millipore); anti-HA (#MMS-101R) (Covance); anti-ATG9A (NB110-56893) (Novus); anti-ATG14 (#PD026) (MBL). Following commercially available fluorescent-labeled secondary antibodies were used: Alexa Fluor 488 donkey anti-rabbit IgG (A21206) and Alexa Fluor 594 donkey anti-mouse IgG (A21203) (Invitrogen). We used antibodies recommended by the manufacturer for the species and application. The antibody specifically recognizing arginine methylated Pontin at R333 and R339 (Pontin-me) was generated by Peptron. Regarding chemicals, hygromycin (H3274), puromycin (P8833), and chloroquine (C6628) were from Sigma-Aldrich. Bafilomycin A1 (#11038) was from Cayman. Rapamycin (R-5000) was from LC Laboratories. EPZ025654 (AOB33871) was from AOBIOUS. EZM2302 (HY-111109) was from MedChemExpress.

### Generation of *Pontin*^*f/f*^ MEFs and cell culture

We generated *Pontin*^*f/f*^ MEFs from *Pontin*-deficient mice by using the 3T3 protocol^17^. HEK293T, HeLa, HepG2, *Carm1* KO MEF, and *Pontin*^*f/f*^ MEF cells were cultured at 37 °C in Dulbecco’s modified Eagle’s medium (DMEM) containing 10% fetal bovine serum (FBS) and antibiotics in a humidified incubator with 5% CO_2_. *Pontin*^*f/f*^ MEFs were infected with Flag-tagged Pontin WT, RK, or RA lentiviruses and selected with hygromycin for 7 days to generate stably expressing cell lines. All cell lines used in the study were regularly tested for mycoplasma contamination. For glucose/amino acid starvation, cells were washed with PBS, then incubated with glucose/amino acid-free DMEM supplemented with 10% dialyzed FBS. Treatment of EPZ025654 (100 nM) or EZM2302 (100 nM) was conducted in cells for 96 h. Transfection was performed with Turbofect (Fermentas) or Lipofectamine 3000 (Invitrogen).

### Identification of binding proteins for CARM1 by affinity purification

CARM1-binding proteins were affinity-purified from extracts of HEK293T cells stably expressing Flag-tagged CARM1. As a negative control, a mock purification from HEK293T cells stably expressing an empty vector was performed. The CARM1-binding proteins were immunoprecipitated using Flag-M2 affinity gel (100 μl of 50% slurry) (Sigma) and washed with buffer containing 20 mM Tris–HCl (pH 7.9), 15% Glycerol, 1 mM EDTA, 1 mM dithiothreitol (DTT), 0.2 mM PMSF, 0.05% Nonidet P40, and 150 mM KCl to remove non-specific contaminants, and the bound materials were eluted by competition with the Flag peptide (0.2 mg/ml). The bound proteins were resolved by sodium dodecyl sulfate-polyacrylamide gel electrophoresis (SDS–PAGE) and prepared for LC–MS/MS analysis.

### Bacterial expression and GST-pulldown assay

GST-tagged constructs were transformed in Rosetta *E. coli* strain and purified with glutathione beads (GE Healthcare). His-tagged Pontin was transformed in M15[pREP4] *E. coli* strain and purified with Ni-NTA beads (Qiagen). In vitro translated Pontin and Reptin were generated using TNT Quick Coupled Transcription/Translation system (Promega) according to the manufacturer’s protocol. Purified proteins and in vitro translated proteins were diluted in binding buffer (125 mM NaCl, 20 mM Tris [pH 7.5], 10% glycerol, 0.1% NP-40, and 0.5 mM DTT supplemented with protease inhibitors) for GST-pulldown assays. Samples were then washed four times with dilution buffer and boiled with SDS sample buffer for immunoblot analysis.

### In vitro methylation assay

In vitro methylation assays were performed by incubating bead-bound His-Pontin or GST-Pontin with recombinant CARM1 (Active Motif, 31347) proteins or HA-CARM1 WT or R169A mutant proteins purified from HEK293T cells. The reaction was carried out in PBS buffer at 30 °C for 3 h, terminated by adding 5× sampling buffer, and boiled for 5 min. The reaction mixtures were then run by SDS–PAGE and transferred from the gel to PVDF membrane using a semi-dry electroblotter. Immunoblotting against anti-Rme2a or Pontin-me antibodies was performed to detect arginine methylated Pontin.

### In vitro methylation assay using ^3^H-SAM

In vitro methylation assays were performed by incubating GST-Pontin and recombinant CARM1 (Active Motif, 31347) proteins in methylation buffer (50 mM Tris–HCl pH 8.5, 20 mM KCl, 10 mM MgCl_2_, 10 mM β-mercaptoethanol, and 250 mM sucrose) with 1 μCi of ^3^H-SAM at 30 °C overnight. The reaction buffer was removed and a 2× sampling buffer was added. The reaction mixtures were boiled for 10 min, then run by SDS–PAGE and analyzed by autoradiography.

### Immunodot blot assay

GST-FOXO3a proteins were purified using Glutathione beads in *E. coli*. Non-methylated Pontin peptide and methylated Pontin peptide were spotted on a membrane. After air dry, the membrane was blocked with 5% milk in the PBS-T buffer for 1 h at RT. The membrane was incubated with purified GST-FOXO3a proteins (1 μg/ml) in the protein-binding buffer (100 mM NaCl, 20 mM Tris–HCl [pH 7.6], 10% glycerol, 0.1% Tween-20, 2% skim milk powder, and 1 mM DTT), followed by conjugation with peptides on the membrane. After binding of GST-FOXO3a to Pontin peptides, immunoblot analysis was performed using an anti-GST antibody.

### Lentivirus construction and production

3× Flag-Pontin WT, RK, and RA mutant were cloned into pLVX vector, and lentiviral shRNA constructs were cloned into pLKO.1 vector. Lentiviral constructs were co-transfected with packaging vectors (psPAX2 and VSV-G) in HEK293T cells and the supernatants were collected and filtered through a 0.45-μm membrane 48 h post-transfection. For the concentration of lentivirus, Lenti-X concentrator was used according to the manufacturer’s instructions (Clontech). Hygromycin selection was performed for 10 days post-infection, and puromycin selection was performed for 36 h post-infection. The targeting sequence of shRNA is as follows. Tip60; 5′-GCAACGCCACTTGACCAAATG-3′, Pontin; 5′-GTGGCGTCATAGTAGAATTA A-3′, FOXO1/3/4; 5′-CTGTGTGCCCTACTTCAAGGA-3′.

### Preparation for whole-cell lysates and subcellular fractionation

All cells were briefly rinsed with ice-cold PBS before collection. For whole-cell lysates, the cells were resuspended in RIPA buffer (150 mM NaCl, 1% Triton X-100, 1% Sodium deoxycholate, 0.1% SDS, 50 mM Tris–HCl [pH 7.5], and 2 mM EDTA) supplemented with protease inhibitors and sonicated using a Branson Sonifier 450 at output 3 and a duty cycle of 30 for 5 pulses. For cytosolic and nuclear fractions, cells were lysed in harvest buffer (10 mM HEPES [pH 7.9], 50 mM NaCl, 0.5 M sucrose, 0.1 mM EDTA, 0.5% Triton X-100, DTT, PMSF, and protease inhibitors), incubated on ice for 5 min and spun at 100 × *g* for 10 min at 4 °C. The supernatant (cytosolic fraction) was removed to a separate tube. The nuclear pellet was rinsed twice with 500 μl of buffer A (10 mM HEPES [pH 7.9], 10 mM KCl, 0.1 mM EDTA, and 0.1 mM EGTA) and spun down at 100 × *g* for 10 min at 4 °C. The supernatant was discarded and the pellet (nuclear fraction) was resuspended in RIPA buffer. All lysates were quantified by the Bradford method and analyzed by SDS–PAGE.

### Autophagic vacuole staining

Autophagic vacuoles were stained by fluorescence microscopy using the Cyto-ID autophagy detection kit (Enzo Life Sciences, ENZ-51031). Cells grown on coverslips at a density of 2 × 10^4^ cells were incubated with a medium containing Cyto-ID green detection reagent (1:500) and Hoechst 33342 (1:1000) at 37 °C for 30 min. After staining, the cells were washed with PBS and then fixed with 2% paraformaldehyde in PBS at RT for 10 min. Cells were mounted and visualized under a confocal microscope (Zeiss, LSM700).

### Immunofluorescence

Cells grown on coverslips at a density of 5 × 10^4^ cells were washed with PBS, then fixed with 2% paraformaldehyde in PBS at RT for 10 min. Fixed cells were permeabilized with 0.5% Triton X-100 in PBS (PBS-T) at RT for 10 min. Blocking was performed with 3% bovine serum in PBS-T for 1 h. For staining, cells were incubated with antibodies at RT for 2 h, followed by incubation with fluorescent-labeled secondary antibodies for 1 h. Cells were mounted and visualized under a confocal microscope (Zeiss, LSM700). For autophagy studies, MEFs were transfected with GFP-LC3 and sub-cultured onto coverslips. The following day, cells were incubated with either complete media or glucose-starved media for 18 h.

### Luciferase assay

MEFs were transiently transfected with the reporter construct bearing 6× canonical FOXO response element (6× DBE-luciferase). Luciferase activity was measured 36 h post-transfection and normalized by β-galactosidase expression.

### Quantitative RT-PCR

Total RNAs were extracted using Trizol (Invitrogen) and reverse transcription was performed from 2.5 μg of total RNAs using the SuPrimeScript cDNA Synthesis Kit (GeNet Bio). The abundance of mRNA was detected by an ABI prism 7500 system or BioRad CFX384 with SYBR TOPreal qPCR 2× PreMix (Enzynomics). The quantity of mRNA was calculated using the ddCt method and HPRT, GAPDH, and β-actin were used as controls. All reactions were performed as triplicates. The following mouse primers were used.

*β-actin*; forward (fwd) 5′-TAGCCATCCAGGCTGTGCTG-3′,

reverse (rev) 5′-CAGGATCTTCATGAGGTAGTC-3′;

*Gapdh*; fwd 5′-CATGGCCTTCCGTGTTCCTA-3′,

rev 5′-CCTGCTTCACCACCTTCTTGA-3′;

*Hprt*; fwd 5′-GCTGGTGAAAAGGACCTCTCG-3′,

rev 5′-CCACAGGACTAGAACACCTGC-3′;

*Map1lc3b*; fwd 5′-CACTGCTCTGTCTTGTGTAGGTTG-3′,

rev 5′-TCGTTGTGCCTTTATTAGTGCATC-3′;

*Sirt1*; fwd 5′-GATACCTTGGAGCAGG TTGC-3′,

rev 5′-CTCCACGAACAGCTTCACAA-3′;

*Bnip3*; fwd 5′-GCTCCCAGACACCA CAAGAT-3′,

rev 5′-TGAGAGTAGCTGTGCGCTTC-3′;

*Ulk1*; fwd 5′-GCTCCGGTGACTT ACAAAGCTG-3′,

rev 5′-GCTGACTCCAAGCCAAAGCA-3′;

*Ctns*; fwd 5′-ATGAGGAGG AATTGGCTGCTT-3′,

rev 5′-ACGTTGGTTGAACTGCCATTTT-3′;

*Cln5*; fwd 5′-CATGAC GCCGTGGGATTCA-3′,

rev 5′-TGACAGTGTCCCGTTTTCCTT-3′;

*Vps33a*; fwd 5′-AGACGTCACCACTTCTGAAGAC-3′,

rev 5′-TGCTTTTGGGCAATGCAGTC-3′;

*Mcoln1*; fwd 5′-ATCTTGATTGCCACGTTGCG-3′,

rev 5′-TAGAACGATCCAGCCACAGAAG-3′;

*Atg2a*; fwd 5′-AACAGAGGCAACATCCAACG-3′,

rev 5′-AATCGCCACTTGAGAGCATG-3′;

*Atg9a*; fwd 5′-TGCCAACAAGATGGTGAACC-3′,

rev 5′-AGACTTGGGCAGGCAAAAAG-3′;

*Atg13*; fwd 5′-ATTTGCACCCGCTCATCATC-3′,

rev 5′-AGGGCCTTCTTTGCTTCATG-3′;

*Atg14*; fwd 5′-AAGCATGGTGAGCAAGCTTG-3′,

rev 5′-ATGCTAATGCTGGTGTCACC-3′.

*Sqstm1*; fwd 5′-ATGTGGAACATGGAGGGAAGA-3′,

rev 5′-GGAGTTCACCTGTAGATGGGT-3′.

*Sesn2*; fwd 5′-TAGCCTGCAGCCTCACCTAT-3′,

rev 5′- GATTTTGAGGTTCCGTTCCA-3′.

The following human primers were used.

*ACTB*; fwd 5′-ATTGCCGACAGGATGCAGAA-3′,

rev 5′-ACATCTGCTGGAAGGTGGACAG-3′;

*GAPDH*; fwd 5′-CGACCACTTTGTCAAGCTCA-3′,

rev 5′-AGGGGAGATTCAGTGTGGTG-3′;

*HPRT*; fwd 5′-TGACACTGGCAAAACAATGCA-3′,

rev 5′-GGTCCTTTTCACCAGCAAGCT-3′;

*MAP1LC3B*; fwd 5′-AAGGCGCTTACAGCTCAATG-3′,

rev 5′-ACACCTCTGAGATTGGTGTGG-3′;

*SIRT1*; fwd 5′-ACATTTTCCATGGCGCTGAG-3′,

rev 5′-TGGCATGTCCCACTATCACTG-3′;

*ULK1*; fwd 5′-TCGCCGTCAAGTGCATTAAC-3′,

rev 5′-AGGGCCACGATGTTTTCATG-3′;

*CTNS*; fwd 5′-TGATCATCATCGTGCAGTGC-3′,

rev 5′-TGGTGACAAATGCGAAGAGC-3′;

*MCOLN1*; fwd 5′-TGTTTGACGTGGTGGTCATC-3′,

rev 5′-ACAAACTCGTTCTGCAGCAG-3′.

### ChIP assay

The ChIP assays were conducted as previously described^16^. In brief, cells were crosslinked with 1% formaldehyde for 10 min at room temperature. After glycine quenching, the cells were collected and lysed in a buffer containing 50 mM Tris–HCl (pH 8.1), 10 mM EDTA, 1% SDS, supplemented with a complete protease inhibitor cocktail (Roche). After sonication, chromatin extracts containing DNA fragments with an average of 250 bp were then diluted ten times with dilution buffer containing 1% Triton X-100, 2 mM EDTA, 150 mM NaCl, and 20 mM Tris–HCl (pH 8.1) with complete protease inhibitor cocktail and subjected to immunoprecipitations overnight at 4 °C. Immunocomplexes were captured by incubating 40 μl of protein A/G Sepharose for 2 h at 4 °C. Beads were washed with TSE I buffer (0.1% SDS, 1% Triton X-100, 2 mM EDTA, 20 mM Tris–HCl (pH 8.1) and 150 mM NaCl), TSE II buffer (0.1% SDS, 1% Triton X-100, 2 mM EDTA, 20 mM Tris–HCl (pH 8.1) and 500 mM NaCl), buffer III (0.25 M LiCl, 1% NP-40, 1% deoxycholate, 10 mM Tris–HCl (pH 8.1) and 1 mM EDTA), three times TE buffer (10 mM Tris–HCl (pH 8.0) and 1 mM EDTA) and eluted in elution buffer (1% SDS and 0.1 M NaHCO_3_). The supernatant was incubated overnight at 65 °C to reverse crosslink and then digested with RNase A for 2 h at 37 °C and proteinase K for 2 h at 55 °C. ChIP and input DNA were then purified and analyzed for qRT–PCR analysis or used for constructing sequencing libraries.

The following commercially available antibodies were used in ChIP assay: anti-Pontin (SAB4200194) (Sigma-Aldrich); anti-CARM1 (#3379), anti-phospho-FOXO3a S413 (#8174), and anti-Tip60 (#12058) (Cell Signaling Technology); anti-FOXO3a (ab12162) and anti-H3R17me2 (ab8284) (Abcam); anti-acetyl-Histone H4 (06-866) (Millipore); anti-IgG (sc-2025) (Santa Cruz Biotechnology). The antibody specifically recognizing arginine methylated Pontin at R333 and R339 (Pontin-me) was generated by Peptron. Each antibody used 1 μg per chromatin IP reaction.

The ChIP assays were conducted with the following primers.

Map1lc3b (FOXO RE, −1.2 kb); fwd 5′-CATGCCTTGGGACACCAGAT-3′,

rev 5′-ACCTTCTTCAAGTGCTGTTTGT-3′;

Map1lc3b (CLEAR motif); fwd 5′-AGCCAGTGGGATATTGGTCT-3′,

rev 5′-AGAGCCTGCGGTACCCTAC-3′;

Map1lc3b (FOXO RE, −11.3 kb); fwd 5′-AAACTTAGGACGCCCAGCTG-3′,

rev 5′-GTAAAACCCCTTCGTTCTACCC-3′;

Map1lc3b (FOXO RE, −5 kb); fwd 5′-CCTCGACGATCTTGAGCAATTC-3′,

rev 5′-AAACTATTGGCCAGCGATGC-3′;

Bnip3; fwd 5′-GCCCTCGTATAACCTTAGCA-3′,

rev 5′-TGGGTCAGGTCACTAGAAGC-3′;

Sirt1 (FOXO RE); fwd 5′-TTTAACTGCCCCCTTTCCAT-3′,

rev 5′-GCGAGACCAGTCTCCAAAAC-3′;

Ctns (FOXO RE, +38.9 kb); fwd 5′-ATAGAAGCGGTGGAAAAGCC-3′,

rev 5′-AGTGCAGGAAGTAAAGGAGGAG-3′;

Ctns (FOXO RE, +23.1 kb); fwd 5′-TTCCAGACGAGCCACTTCTG-3′,

rev 5′-TTCACTGTGTACCGGCATTG-3′.

Ctns (CLEAR motif); fwd 5′-CCTCTGGTAGCGTAGGTTGC-3′,

rev 5′-GCTTTTGGTGAGGTCTGTCC-3′.

Atg14 (FOXO RE); fwd 5′-AAAAACCCCACATTTATCTTTGA-3′,

rev 5′-CAGGCTAACACTCCCGACAT-3′.

Atg14 (CLEAR motif); fwd 5′-AGAAACTCTGGTGGGTGGTG-3′,

rev 5′-TGGAAAAGAGAATGGAGCTG-3′.

### RNA-seq analysis

RNA-seq libraries were prepared using the TruSeq RNA sample prep kit v2 (Illumina) according to the manufacturer’s instructions. RNA-seq libraries were paired-end sequenced on an Illumina HiSeq 4000 (Macrogen). RNA-seq data were mapped using Tophat package^42^ against the mouse genome (mm9). Differential analysis was done via EdgeR package^43^. Differentially regulated genes were identified using a false discovery rate (FDR) cut-off of 1 × 10^−5^ for Pontin WT against Pontin RA under the normal condition as well as glucose-starved condition. Hierarchical clustering analysis was done using the gene expression values from all conditions. Ward’s criterion for genes with 1 − (correlation coefficient) was used as a distance measure. A clustering heatmap was drawn using a *z*-score that is scaled across samples for each gene. Functional enrichment analysis of GOBPs and KEGG pathways was performed using a DAVID software^44,45^. Gene set enrichment analysis was performed using a GSEA (version 3.0) software^46,47^. The phenotype label for GSEA was set WT_normal: WT_Glc starv.: RA_normal: RA_Glc starv. = 1: 3: 1: 1.7, then the Pearson correlation coefficient was calculated per gene for ranking. Gene sets were obtained from the online available Molecular Signature Database (MSigDB) v6.2 of the Broad Institute (http://software.broadinstitute.org/gsea/msigdb/index.jsp).

### ChIP-seq analysis

ChIP-seq libraries were prepared for sequencing using the TruSeq DNA Sample Prep Kit, according to the manufacturer’s instructions. ChIP-seq libraries were paired-end sequenced on an Illumina platform (MACROGEN). ChIP-seq reads were aligned to the mouse reference genome (GRCm38/mm10) using Bowtie2. For peak calling for methylated Pontin, we used Homer (v4.7.2). Methylated Pontin upon glucose starvation was compared against control input. We used Pontin-me (specifically recognizing arginine methylated Pontin at R333 and R339) antibodies for ChIP-seq.

### Chromosome conformation capture (3C) assay

The 3C assays were performed as described previously with little modifications^48^. In brief, formaldehyde was added to the media to a final concentration of 1% at room temperature for 10 min. Glycine was added to stop the cross-linking reaction to a final concentration of 0.125 M. After washing with PBS, cells were lysed in cold lysis buffer (10 mM Tris–HCl, pH 7.5; 10 mM NaCl; 0.2% NP-40; and 1× complete protease inhibitor). After centrifugation, collected nuclei were resuspended with 1.2× restriction enzyme buffer (buffer H; Takara) with 0.3% (w/v) SDS and incubated at 37 °C for 1 h. 2% (v/v) Triton X-100 was added at 37 °C for 1 h while shaking at 900 r.p.m. Chromatin digestion was performed by 400 U of restriction enzyme (Bgl II; Takara) was added overnight at 37 °C. The restriction enzymes were heat-inactivated at 65 °C for 20 min and diluted by the addition of 6 ml of ligation buffer (66 mM Tris–HCl, pH 7.5; 5 mM DTT; 5 mM MgCl_2_; and 1 mM ATP). The chromatin was ligated by the addition of 100 U of T4 DNA ligase (New England BioLabs) at 16 °C for 4 h followed by 30 min at room temperature. Ligated chromatin was incubated at 65 °C overnight to reverse crosslink. The DNA was purified by phenol–chloroform extraction and ethanol precipitation. The following PCR program was used: 95 °C for 10 min, followed by 35 cycles of 95 °C for 10 s, 61 °C for 10 s, and 72 °C for 10 s. The following mouse primers were used.

*Map1lc3b* promoter fwd (P) 5′-TCTAGGCCTTCAGTTTGGACCCTTGTTTGC-3′;

*Map1lc3b* enhancer_1 fwd (1) 5′-TTAGCAAGATAGTTGGTCTTAGCCAGGTG-3′;

*Map1lc3b* enhancer_2 fwd (2) 5′-AGAGCCTTCTGTGCTAGCTGAAGAACCTG-3′.

### Statistics and reproducibility

All experiments were performed independently at least three times. Statistical analysis was done using GraphPad Prism v5. Student’s *t*-test was used for comparison between two groups. ANOVA analysis with post hoc testing was used for multiple sample comparisons and discrimination of significance relationships. A *p*-value of <0.05 was considered statistically significant.

### Reporting summary

Further information on research design is available in the Nature Research Reporting Summary linked to this article.

## Supplementary information

Supplementary Information

Reporting Summary

## Data Availability

RNA-seq and ChIP-seq data that support the findings of this study have been deposited in the Gene Expression Omnibus under the accession number GSE130069 and GSE157952, respectively. 3D structure of the Pontin from Matias et al.^49^ and FOXO3a from Wang et al.^50^ are available from the Protein Data Bank under accession code PDB ID: PDB “2C9O” [https://www.rcsb.org/structure/2c9o] and PDB “2LQH” [https://www.rcsb.org/structure/2LQH]. Gene sets were obtained from the online available Molecular Signature Database (MSigDB) v6.2 of the Broad Institute [http://software.broadinstitute.org/gsea/msigdb/index.jsp]. All relevant data presented in this paper are available from the corresponding author upon request. Source data are provided with this paper.

## Source data

Source Data
