# Supplementary material for: Pontin arginine methylation by CARM1 is crucial for epigenetic regulation of autophagy
Source: Nat Commun. 2020 Dec 8;11:6297. doi: 10.1038/s41467-020-20080-9 (PMC7722926; doi:10.1038/s41467-020-20080-9)
Supplement: Supplementary file 1 — Supplementary Information [file 41467_2020_20080_MOESM1_ESM.pdf]

## Supplementary information

**Supplementary information includes 10 Supplementary Figures and 2 Supplementary Tables.**

**Supplementary Figure 1. Pontin is methylated by CARM1 upon glucose starvation, related to Figures 1 and 2.**

**Supplementary Figure 2. Increased Pontin methylation by CARM1 upon rapamycin treatment or amino acid starvation, related to Figure 3.**

**Supplementary Figure 3. Glucose starvation-induced Pontin methylation in various cell lines and its effect on cell proliferation and viability, related to Figure 3.**

**Supplementary Figure 4. RNA-seq analysis with cluster profiling, related to Figure 4.**

**Supplementary Figure 5. Identification of methylated Pontin-dependent target genes by RNA-seq analysis, related to Figure 4.**

**Supplementary Figure 6. Quantitative RT-PCR analysis of methylated Pontin-dependent autophagy and lysosomal genes, related to Figure 4.**

**Supplementary Figure 7. Identifying FOXO3a domains necessary for Pontin binding, related to Figures 5, 6.**

**Supplementary Figure 8. Recruitment of methylated Pontin and Tip60 along with H4 acetylation on the FOXO3 response elements, related to Figure 6.**

**Supplementary Figure 9. Pontin methylation is not involved in regulation of Skp2 by FOXO3a, related to Figure 8.**

**Supplementary Figure 10. CARM1 is recruited on CELAR motif, but not on the FOXO3 response, related to Figure 8.**

**Supplementary Table 1. List of protein and peptide sequences identified by LC-MS/MS analysis, related to Figure 1.**

**Supplementary Table 2. List of genes from GO Term analysis, related to Figure 4.**

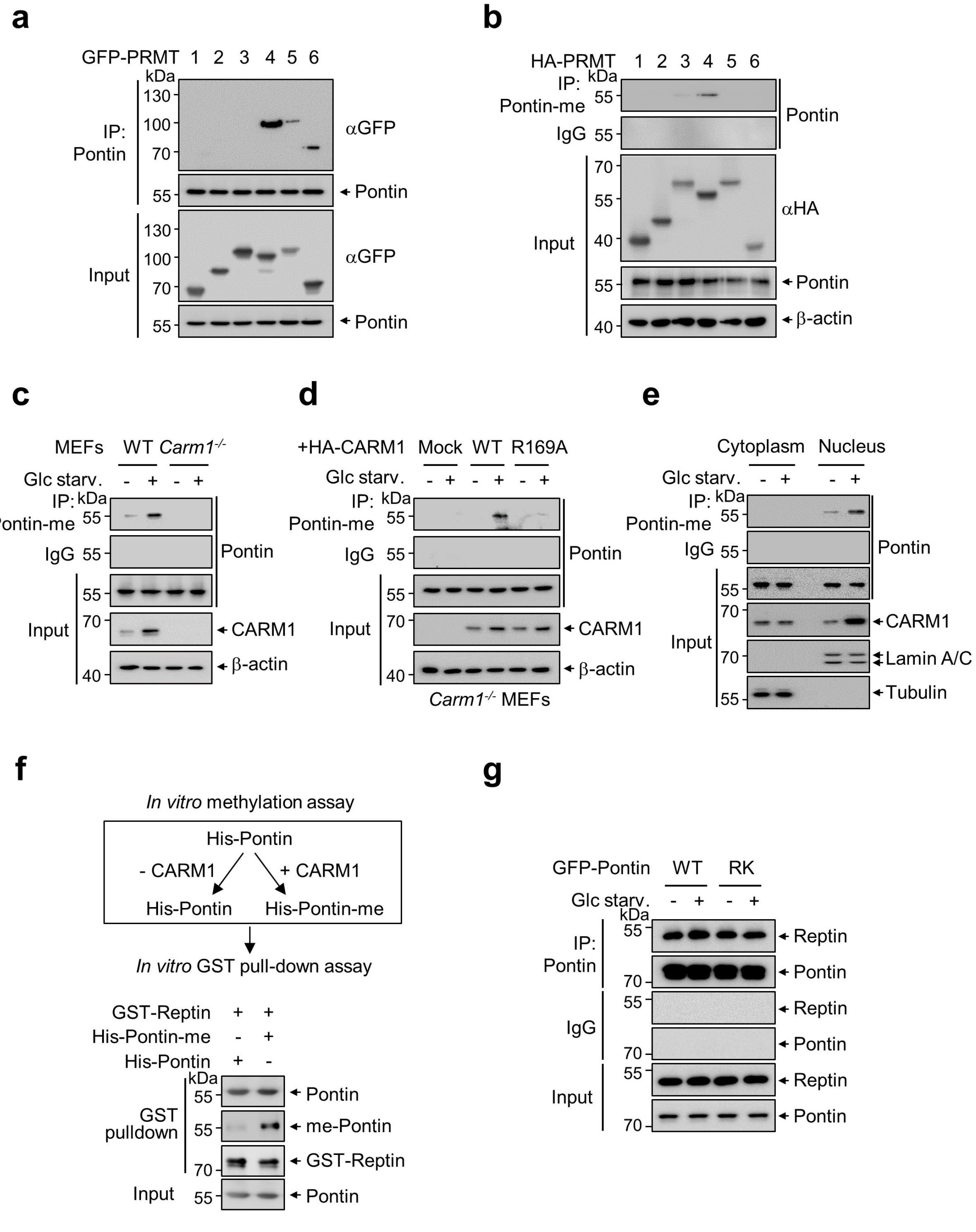
a
GFP-PRMT 1 2 3 4 5 6
kDa
130
100
70
55
IP: Pontin
αGFP
Pontin
Input
αGFP
Pontin
b
HA-PRMT 1 2 3 4 5 6
IP: Pontin-me
IgG
Pontin
Input
αHA
Pontin
β-actin
c
MEFs WT Carm1-/-
Glc starv. - + - +
IP: Pontin-me
IgG
Pontin
Input
CARM1
β-actin
d
+HA-CARM1 Mock WT R169A
Glc starv. - + - + - +
IP: Pontin-me
IgG
Pontin
Input
CARM1
β-actin
Carm1-/- MEFs
e
Cytoplasm Nucleus
Glc starv. - + - +
IP: Pontin-me
IgG
Pontin
Input
CARM1
Lamin A/C
Tubulin
f
In vitro methylation assay
His-Pontin
- CARM1
+ CARM1
His-Pontin
His-Pontin-me
In vitro GST pull-down assay
GST-Reptin + +
His-Pontin-me - +
His-Pontin + -
GST pulldown
Pontin
me-Pontin
GST-Reptin
Input
Pontin
g
GFP-Pontin WT RK
Glc starv. - + - +
IP: Pontin
Reptin
Pontin
IgG
Reptin
Pontin
Input
Reptin
Pontin

**Supplementary Figure 1. Pontin is methylated by CARM1 upon glucose starvation, related to Figures 1 and 2.**

(**a**) Immunoprecipitation assay between Pontin and various arginine methyltransferases including PRMT1 to PRMT6.
**(b)** Pontin methylation was assessed using anti-Pontin-me antibody in cells expressing PRMT1 to PRMT6.
(**c**) Immunoprecipitation assay with anti-Pontin-me antibody in WT or *Carm1*$^{-/-}$ MEFs following glucose starvation.
(**d**) Pontin methylation was assessed in *Carm1*$^{-/-}$ MEFs reconstituted with CARM1 WT or R169A mutant.
(**e**) Pontin methylation was assessed using anti-Pontin-me antibody in HepG2 cells after nuclear and cytoplasmic fractionation.
(**f**) Flowchart of *in vitro* methylation assay to prepare for non-methylated and methylated Pontin (above). GST pulldown assay was performed to examine the binding of Reptin to methylated Pontin or non-methylated Pontin (below).
(**g**) Co-immunoprecipitation assay was performed to detect interaction of Reptin to Pontin WT or RK mutant.
Source data are provided as a Source Data file.

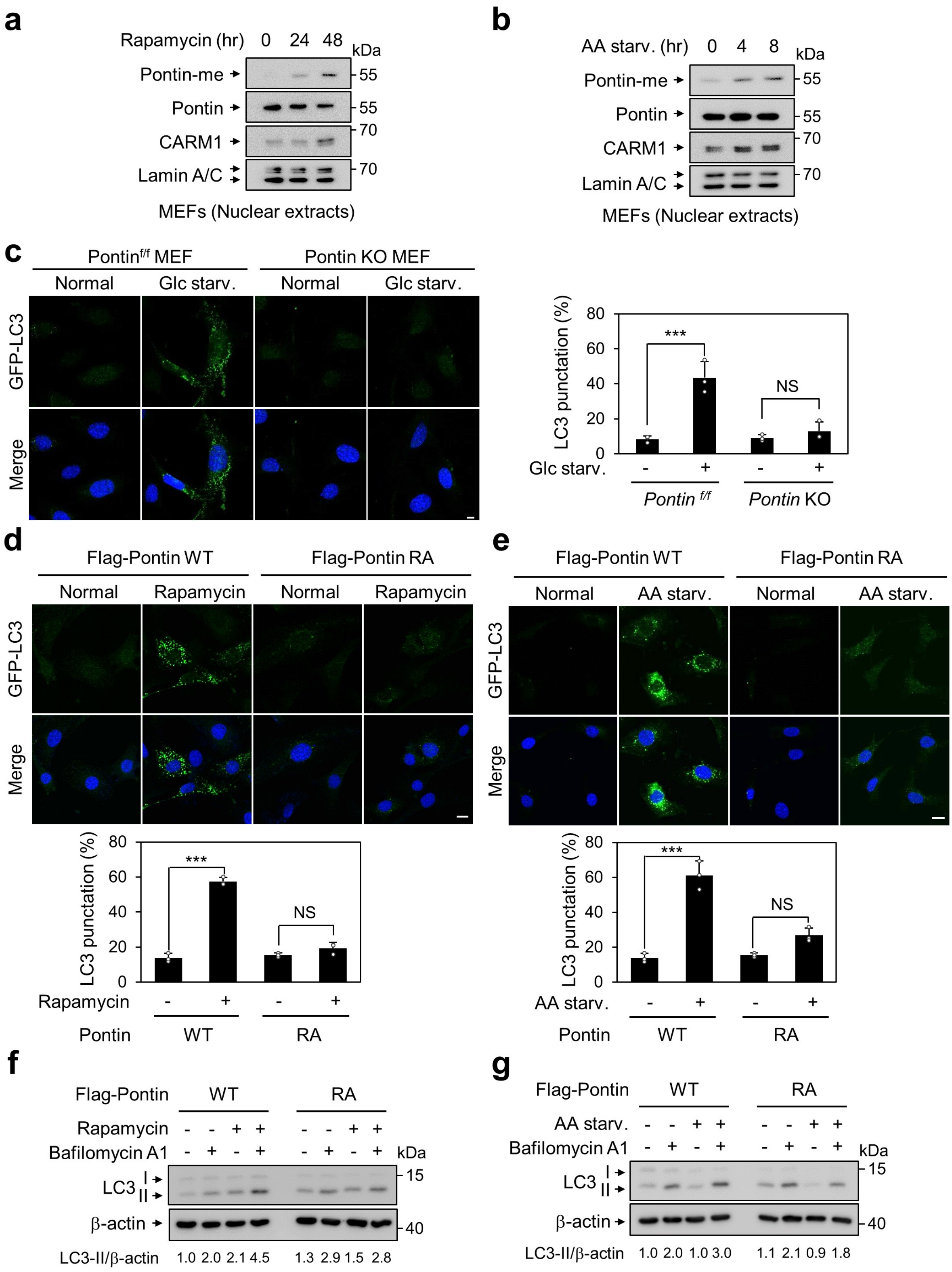
a
Rapamycin (hr) 0 24 48
kDa
Pontin-me
55
Pontin
55
CARM1
70
Lamin A/C
70
MEFs (Nuclear extracts)
b
AA starv. (hr) 0 4 8
kDa
Pontin-me
55
Pontin
55
CARM1
70
Lamin A/C
70
MEFs (Nuclear extracts)
c
Pontin f/f MEF
Pontin KO MEF
Normal
Glc starv.
Normal
Glc starv.
GFP-LC3
Merge
LC3 punctation (%)
80
60
40
20
0
***
NS
Glc starv.
- + - +
Pontin f/f
Pontin KO
d
Flag-Pontin WT
Flag-Pontin RA
Normal
Rapamycin
Normal
Rapamycin
GFP-LC3
Merge
LC3 punctation (%)
***
NS
Rapamycin
- + - +
Pontin
WT
RA
e
Flag-Pontin WT
Flag-Pontin RA
Normal
AA starv.
Normal
AA starv.
GFP-LC3
Merge
LC3 punctation (%)
***
NS
AA starv.
- + - +
Pontin
WT
RA
f
Flag-Pontin WT RA
Rapamycin - - + + - - + +
Bafilomycin A1 - + - + - + - +
kDa
LC3 I II
15
β-actin
40
LC3-II/β-actin 1.0 2.0 2.1 4.5 1.3 2.9 1.5 2.8
g
Flag-Pontin WT RA
AA starv. - - + + - - + +
Bafilomycin A1 - + - + - + - +
kDa
LC3 I II
15
β-actin
40
LC3-II/β-actin 1.0 2.0 1.0 3.0 1.1 2.1 0.9 1.8

**Supplementary Figure 2. Increased Pontin methylation by CARM1 upon rapamycin treatment or amino acid starvation, related to Figure 3.**

(**a**-**b**) Immunoblot analysis of Pontin methylation in nuclear extracts from MEFs in response to rapamycin (100 nM) treatment (**a**) or amino acid (AA) starvation (**b**).
(**c**) Representative confocal images of GFP-LC3 puncta formation upon glucose starvation in Pontin$^{f/f}$ MEFs or Pontin KO MEFs. GFP–LC3 (green); DAPI (blue). Scale bar, 20 μm. The graph shows quantification of LC3-positive punctate cells (right). Bars, mean ± s.e.m.; n=3 with over 50 cells; One-way ANOVA with post hoc Tukey's test. *** p<0.001, NS=Non-Significant.
(**d**-**e**) Representative confocal images of GFP–LC3 puncta formation in MEFs upon rapamycin (100 nM) treatment (**d**) or AA starvation (**e**). GFP–LC3 (green); DAPI (blue). Scale bar, 20 μm. The graph shows quantification of LC3-positive punctate cells (below). Bars, mean ± s.e.m.; n=3 with over 50 cells; One-way ANOVA with post hoc Tukey's test. *** p<0.001, NS=Non-Significant.
(**f**-**g**) Autophagic flux was analyzed in MEFs with or without Bafilomycin A1 (200 nM; 2 hrs) upon rapamycin (100 nM) treatment (**f**) or AA starvation (**g**). The LC3-II/β-actin ratio is indicated (below).
Source data are provided as a Source Data file.

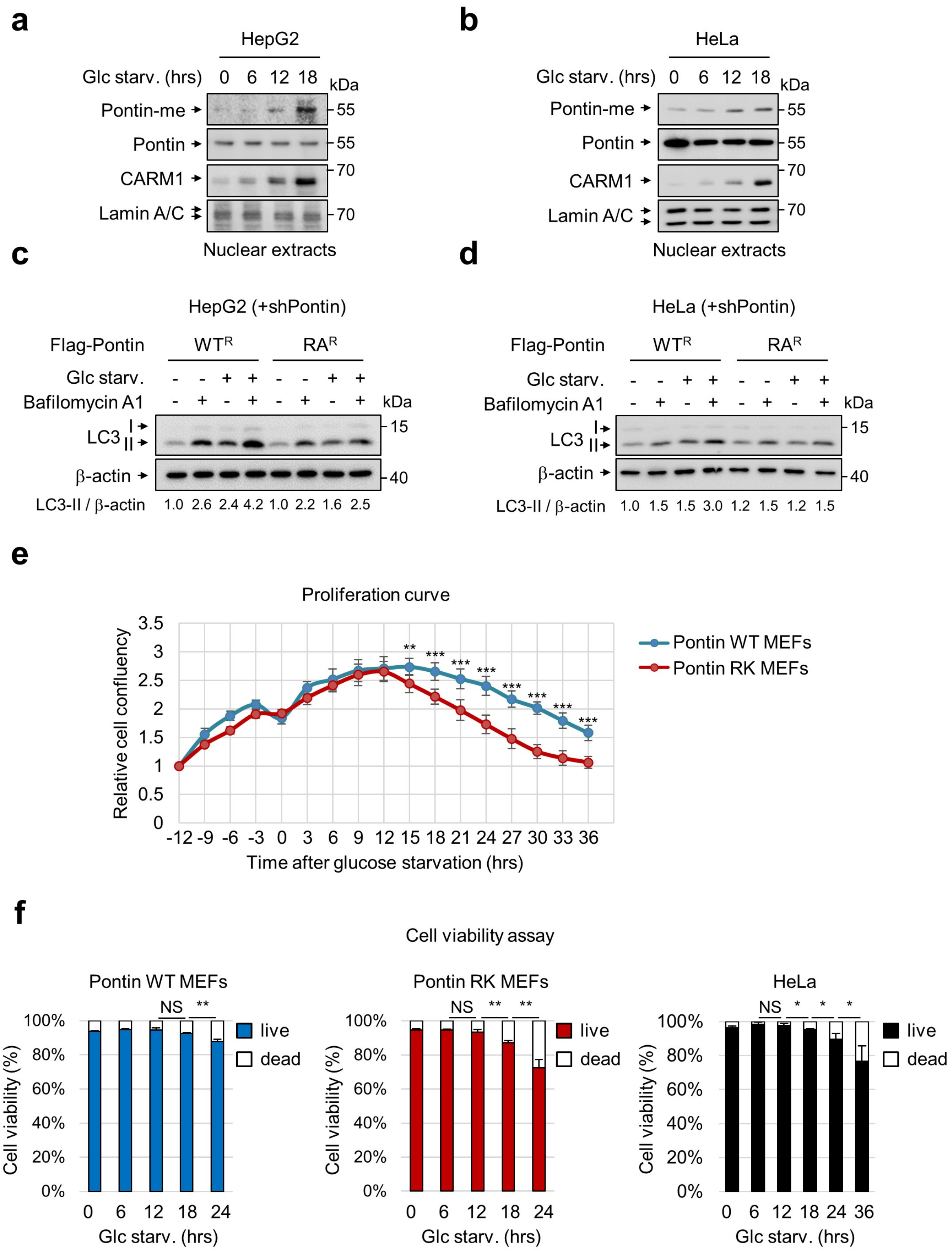

a
HepG2
Glc starv. (hrs) 0 6 12 18 kDa
Pontin-me 55
Pontin 55
CARM1 70
Lamin A/C 70
Nuclear extracts
b
HeLa
Glc starv. (hrs) 0 6 12 18 kDa
Pontin-me 55
Pontin 55
CARM1 70
Lamin A/C 70
Nuclear extracts
c
HepG2 (+shPontin)
Flag-Pontin WTR RAR
Glc starv. - - + + - - + +
Bafilomycin A1 - + - + - + - + kDa
LC3 I II 15
β-actin 40
LC3-II / β-actin 1.0 2.6 2.4 4.2 1.0 2.2 1.6 2.5
d
HeLa (+shPontin)
Flag-Pontin WTR RAR
Glc starv. - - + + - - + +
Bafilomycin A1 - + - + - + - + kDa
LC3 I II 15
β-actin 40
LC3-II / β-actin 1.0 1.5 1.5 3.0 1.2 1.5 1.2 1.5
e
Proliferation curve
Relative cell confluency
Time after glucose starvation (hrs)
Pontin WT MEFs
Pontin RK MEFs
f
Cell viability assay
Pontin WT MEFs
Pontin RK MEFs
HeLa
Cell viability (%)
live
dead
NS
Glc starv. (hrs)

**Supplementary Figure 3. Glucose starvation-induced Pontin methylation in various cell lines and its effect on cell proliferation and viability, related to Figure 3.**

(**a**-**b**) Nuclear extracts from HepG2 (**a**) and HeLa (**b**) cells were subjected to immunoblot analysis with indicated antibodies under glucose starvation.
(**c**-**d**) Autophagic flux was analysed in HepG2 (**c**) and HeLa (**d**) cells transfected with Flag-Pontin WT or RA mutant in the presence or absence of Bafilomycin A1 (200 nM; 2 hrs) upon glucose starvation. The LC3-II/β-actin ratio is indicated (below).
(**e**) Proliferation curve of Pontin WT MEFs and Pontin RA MEFs under glucose starvation. Mean ± s.e.m.; n=6; ** $p<0.01$, *** $p<0.001$. Statistics by two-tailed t-test.
(**f**) Cell viability assays were performed in Pontin WT MEFs, Pontin RA MEFs, or HeLa cells under glucose starvation. Bars, mean ± s.e.m.; n=3; * $p<0.05$, ** $p<0.01$, NS = Non-Significant. Statistics by two-tailed t-test.
Source data are provided as a Source Data file.

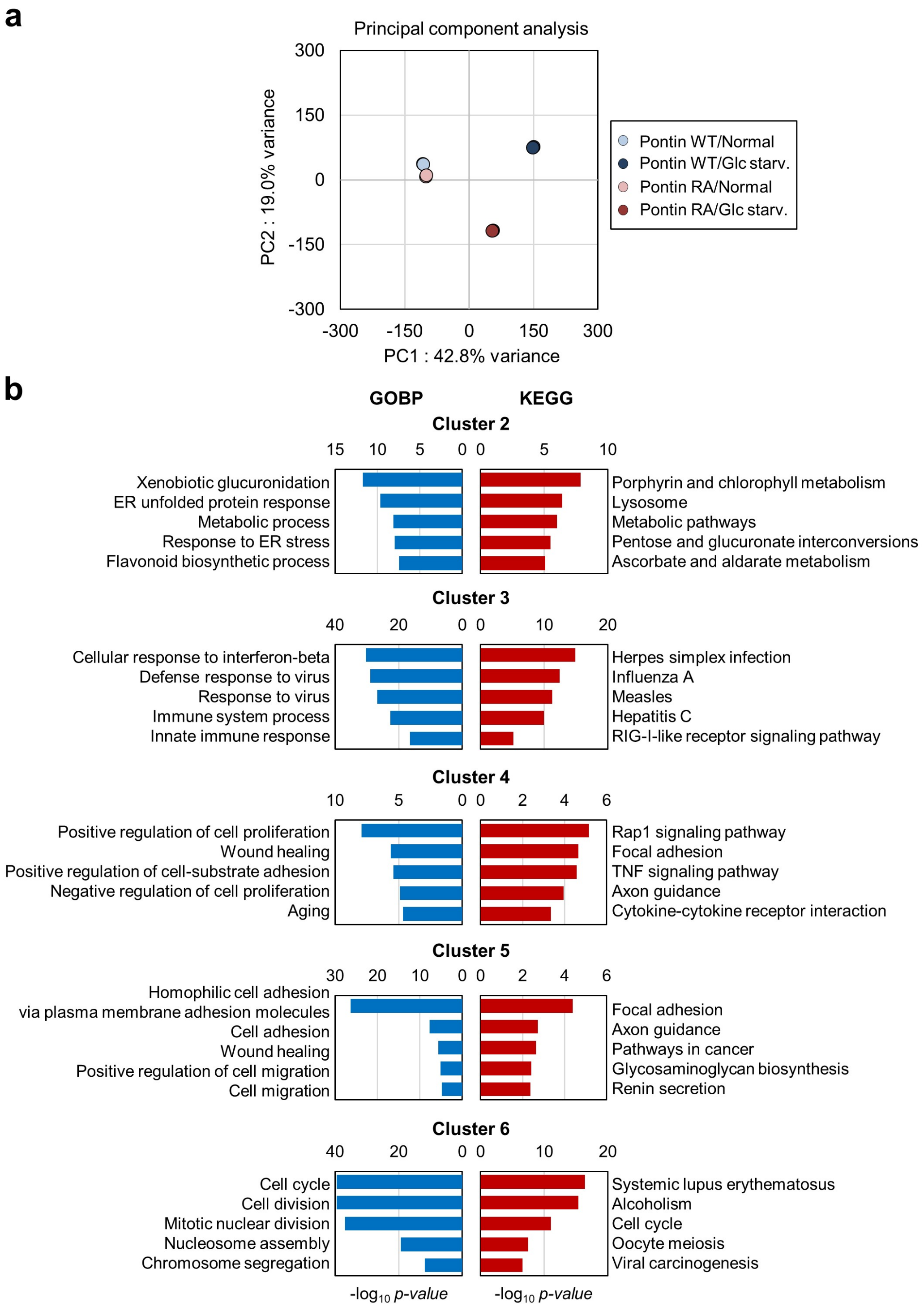


**Supplementary Figure 4. RNA-seq analysis with cluster profiling, related to Figure 4.**

(**a**) Principal component analysis of Pontin WT or RA MEFs under glucose starvation.
(**b**) Gene ontology and KEGG pathway enrichment analyses for the genes in Clusters 2-6 using DAVID software.

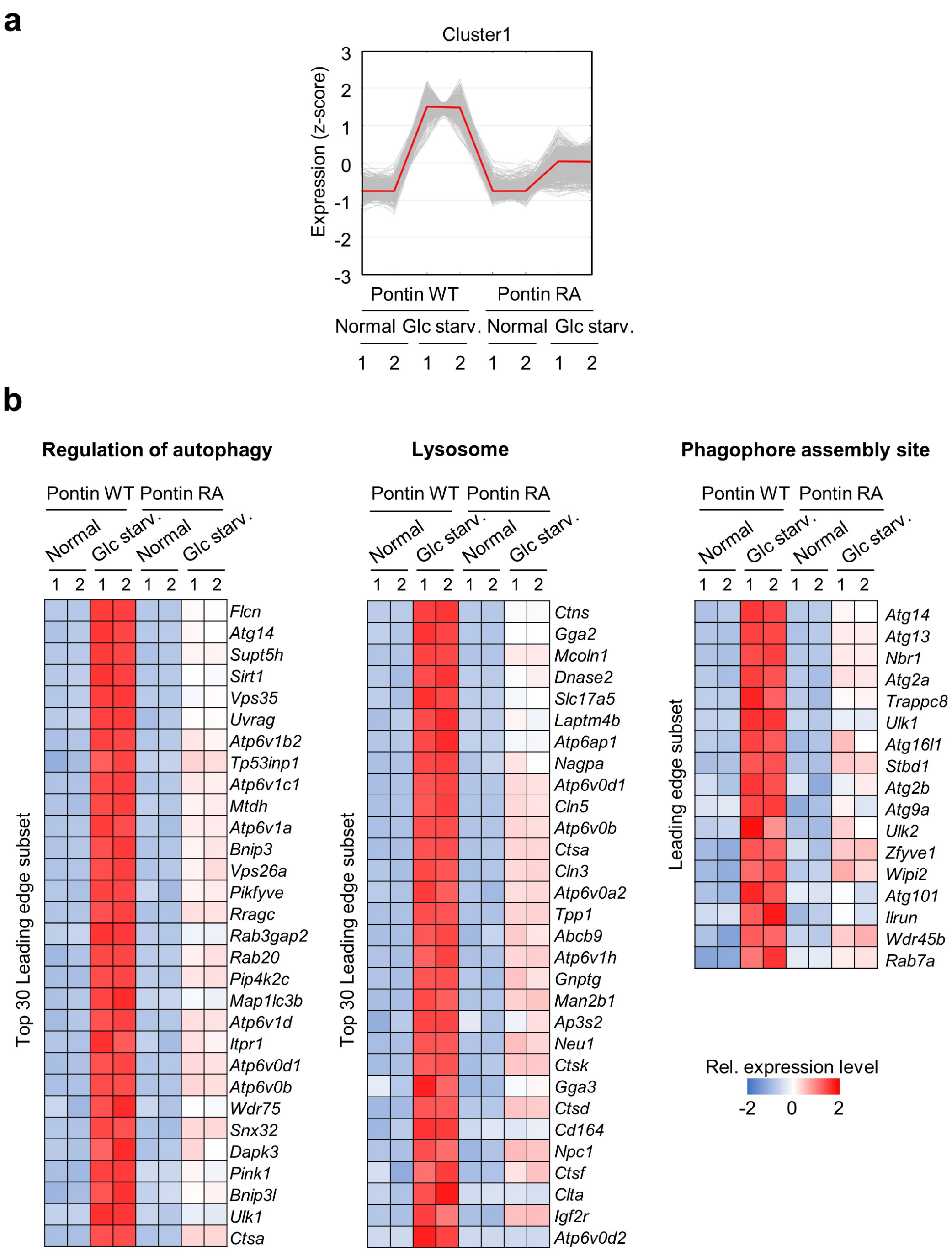


**Supplementary Figure 5. Identification of methylated Pontin-dependent target genes by RNA-seq analysis, related to Figure 4.**

(**a**) Gene expression pattern of cluster 1. Grey lines indicates expression patterns of each cluster 1 genes. Red line indicates median.
(**b**) Representative leading edge subsets contributing to the enrichment of each term. Heatmaps representing expression changes of genes.

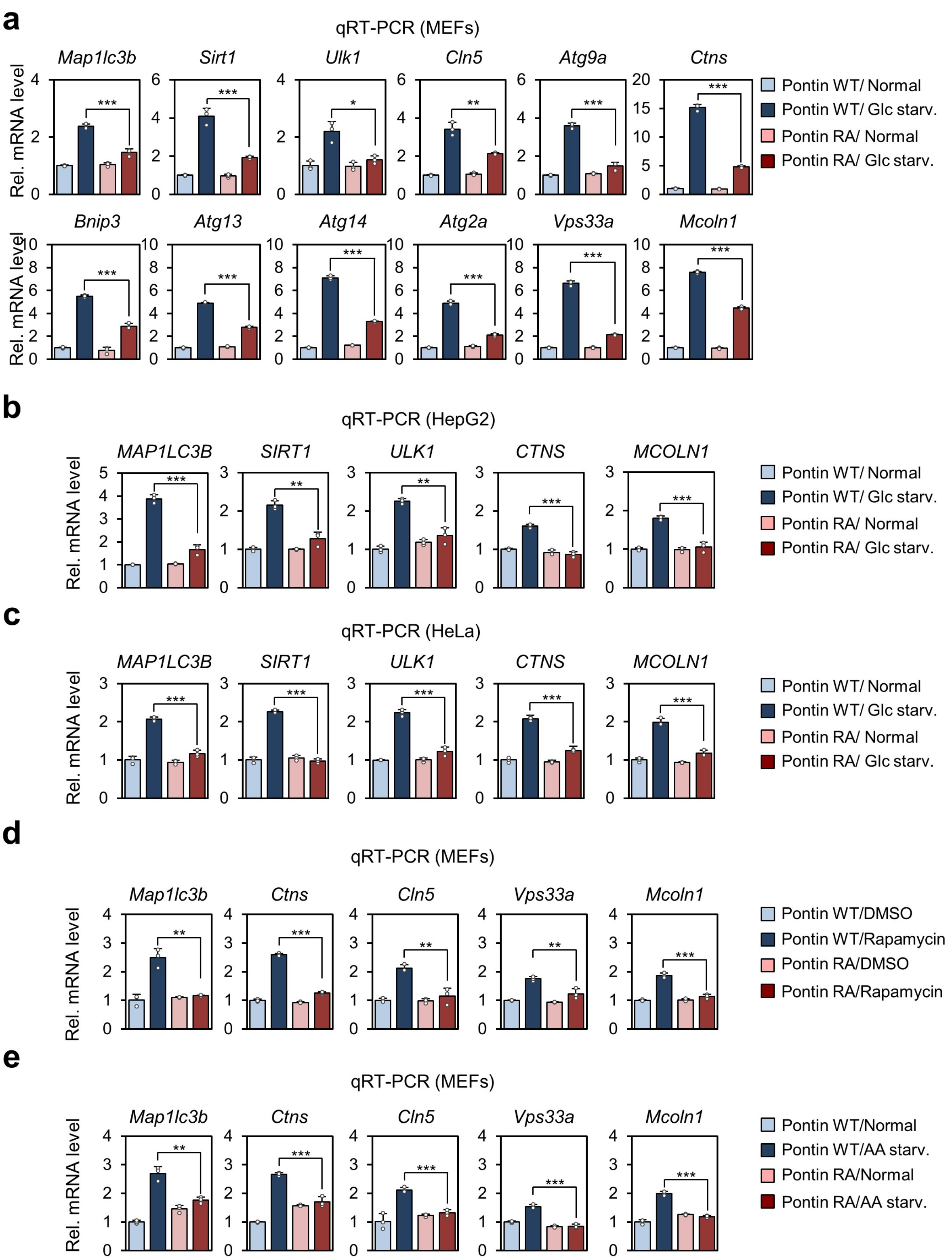
a
qRT-PCR (MEFs)
Map1lc3b
Sirt1
Ulk1
Cln5
Atg9a
Ctns
Rel. mRNA level
Bnip3
Atg13
Atg14
Atg2a
Vps33a
Mcoln1
Pontin WT/ Normal
Pontin WT/ Glc starv.
Pontin RA/ Normal
Pontin RA/ Glc starv.
b
qRT-PCR (HepG2)
MAP1LC3B
SIRT1
ULK1
CTNS
MCOLN1
Rel. mRNA level
Pontin WT/ Normal
Pontin WT/ Glc starv.
Pontin RA/ Normal
Pontin RA/ Glc starv.
c
qRT-PCR (HeLa)
MAP1LC3B
SIRT1
ULK1
CTNS
MCOLN1
Rel. mRNA level
Pontin WT/ Normal
Pontin WT/ Glc starv.
Pontin RA/ Normal
Pontin RA/ Glc starv.
d
qRT-PCR (MEFs)
Map1lc3b
Ctns
Cln5
Vps33a
Mcoln1
Rel. mRNA level
Pontin WT/DMSO
Pontin WT/Rapamycin
Pontin RA/DMSO
Pontin RA/Rapamycin
e
qRT-PCR (MEFs)
Map1lc3b
Ctns
Cln5
Vps33a
Mcoln1
Rel. mRNA level
Pontin WT/Normal
Pontin WT/AA starv.
Pontin RA/Normal
Pontin RA/AA starv.

**Supplementary Figure 6. Quantitative RT-PCR analysis of methylated Pontin-dependent autophagy and lysosomal genes, related to Figure 4.**

(**a**) Quantitative RT-PCR analysis of methylated Pontin-dependent autophagy and lysosomal genes. Bars, mean ± s.e.m.; n=3; *p<0.05, ** p<0.01, *** p<0.001. Statistics by two-tailed t-test.
(**b**-**c**) Quantitative RT-PCR analysis of methylated Pontin-dependent autophagy and lysosomal genes in HepG2 (**b**) and HeLa (**c**) cells transfected with Flag-Pontin WT or RA mutant. Bars, mean ± s.e.m.; n=3; ** p<0.01, *** p<0.001. Statistics by two-tailed *t*-test.
(**d**-**e**) Quantitative RT-PCR analysis of methylated Pontin-dependent autophagy and lysosomal genes in MEFs upon rapamycin (100 nM) treatment (**d**) or amino acid starvation (**e**). Bars, mean ± s.e.m.; n=3; ** p<0.01, *** p<0.001. Statistics by two-tailed *t*-test.

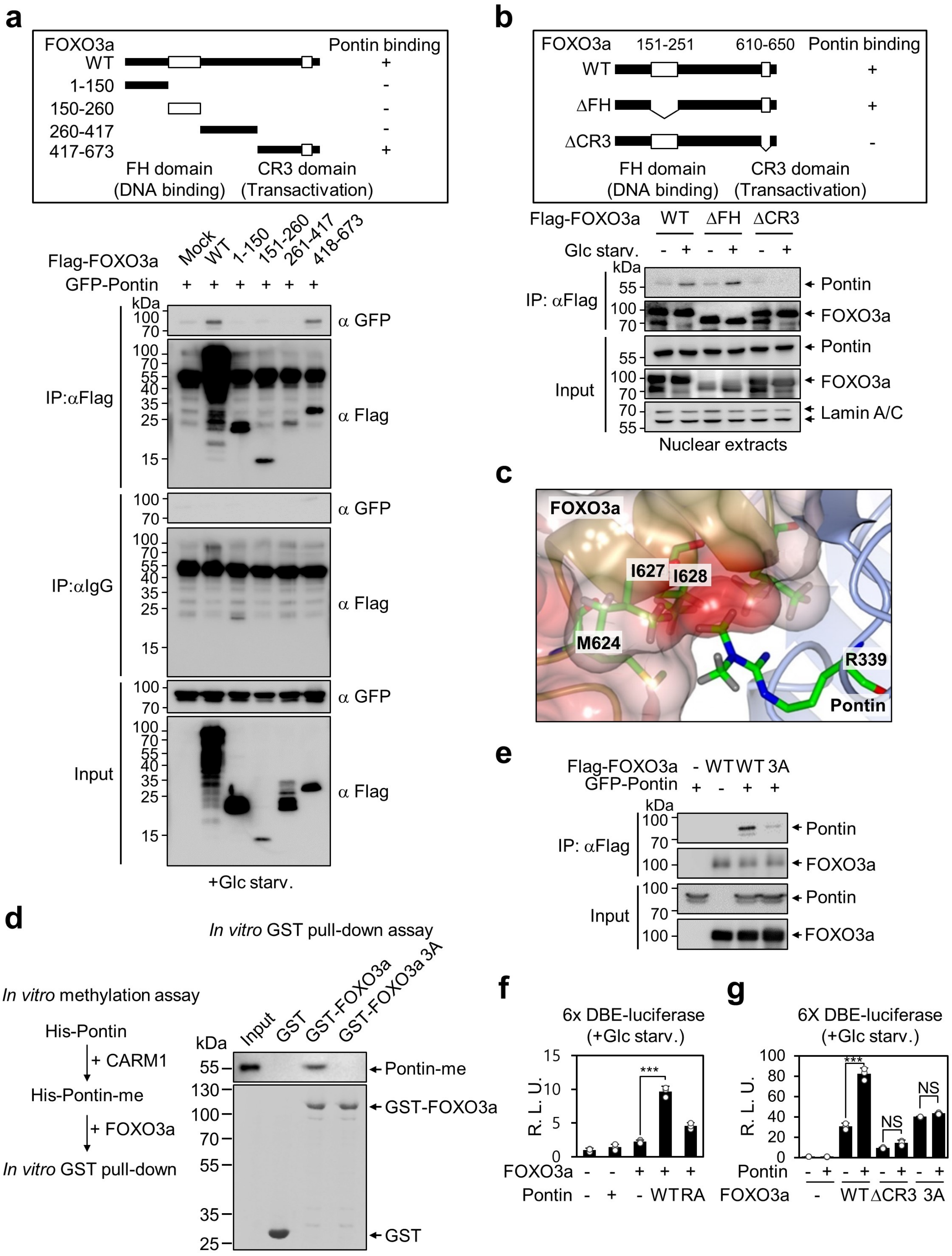

a
FOXO3a
Pontin binding
WT
1-150
150-260
260-417
417-673
FH domain (DNA binding)
CR3 domain (Transactivation)
Flag-FOXO3a
Mock WT 1-150 151-260 261-417 418-673
GFP-Pontin
IP:αFlag
IP:αIgG
Input
α GFP
α Flag
+Glc starv.
b
FOXO3a 151-251 610-650 Pontin binding
WT
ΔFH
ΔCR3
Flag-FOXO3a WT ΔFH ΔCR3
Glc starv.
IP: αFlag
Input
Pontin
FOXO3a
Lamin A/C
Nuclear extracts
c
FOXO3a
I627
I628
M624
R339
Pontin
d
In vitro GST pull-down assay
In vitro methylation assay
His-Pontin
+ CARM1
His-Pontin-me
+ FOXO3a
In vitro GST pull-down
Input GST GST-FOXO3a GST-FOXO3a 3A
Pontin-me
GST-FOXO3a
GST
e
Flag-FOXO3a - WT WT 3A
GFP-Pontin + - + +
IP: αFlag
Input
Pontin
FOXO3a
f
6x DBE-luciferase (+Glc starv.)
R. L. U.
FOXO3a
Pontin
WT RA
g
6X DBE-luciferase (+Glc starv.)
R. L. U.
Pontin
FOXO3a
WT ΔCR3 3A
NS

**Supplementary Figure 7. Identifying FOXO3a domains necessary for Pontin binding, related to Figures 5, 6.**

(**a**-**b**) Schematic illustration of the deletion mutants of FOXO3a (above). Immunoprecipitation assay between Pontin and various truncated FOXO3a proteins upon glucose starvation (below). The numbers refer to amino acids.
(**c**) The modeling of methylated Pontin with FOXO3a. Methylated R339 of Pontin and three amino acid residues (M624, I627, and I628) of FOXO3a forming a hydrophobic pocket are depicted.
(**d**) GST pulldown assay for the binding of methylated Pontin to FOXO3a WT or 3A (M624A, I627A, and I628A) mutant. His-Pontin was purified using Ni-NTA beads. *In vitro* methylation assay was performed by incubating His-Pontin with recombinant CARM1 proteins. The purified methylated Pontin proteins were used to test their interaction with FOXO3a WT and 3A mutant.
(**e**) Immunoprecipitation assay between Pontin and FOXO3a WT or 3A mutant following glucose starvation.
(**f**) Effect of Pontin WT or RA on 6x DBE (FOXO RE)-luciferase reporter. Bars, mean ± s.e.m; n=3; *** $p<0.001$. Statistics by two-tailed *t*-test.
(**g**) Effect of FOXO3a WT, ΔCR3 or 3A mutant on 6x DBE-luciferase reporter. Bars, mean ± s.e.m; n=3; *** $p<0.001$, NS = Non-Significant. Statistics by One-way ANOVA with post hoc Tukey's test.
Source data are provided as a Source Data file.

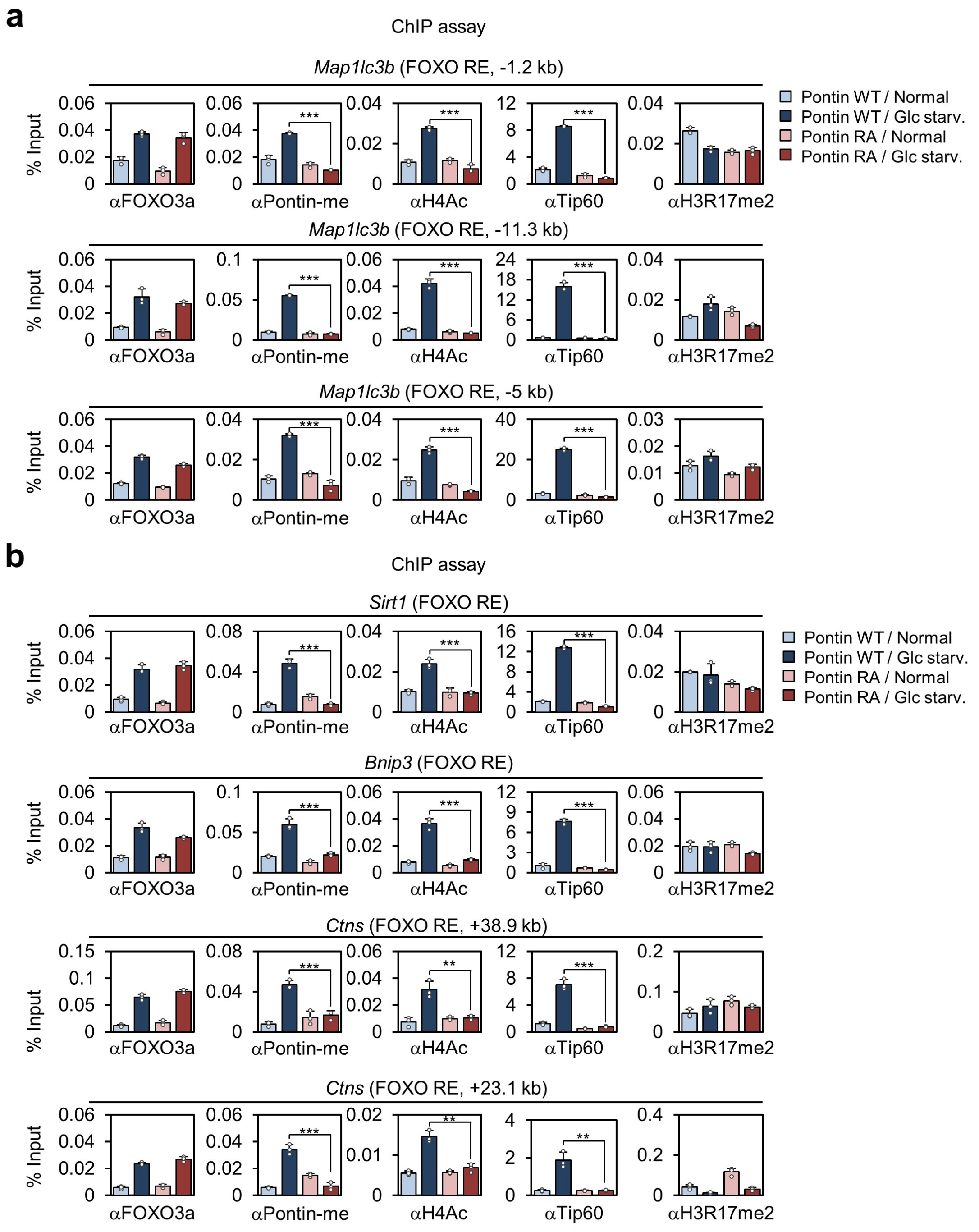


**Supplementary Figure 8. Recruitment of methylated Pontin and Tip60 along with H4 acetylation on the FOXO3 response elements, related to Figure 6.**

(**a**-**b**) ChIP assays were performed using anti-FOXO3a, anti-Pontin-me, anti-H4ac, anti-Tip60, and anti-H3R17me2 antibodies on FOXO REs of *Map1lc3b* (**a**), or *Sirt1*, *Bnip3*, *Ctns* (**b**) genes in Pontin WT or RA-expressing MEFs in the absence or presence of glucose starvation. Bars, mean ± s.e.m; n=3; ** p<0.01, *** p<0.001. Statistics by two-tailed *t*-test.

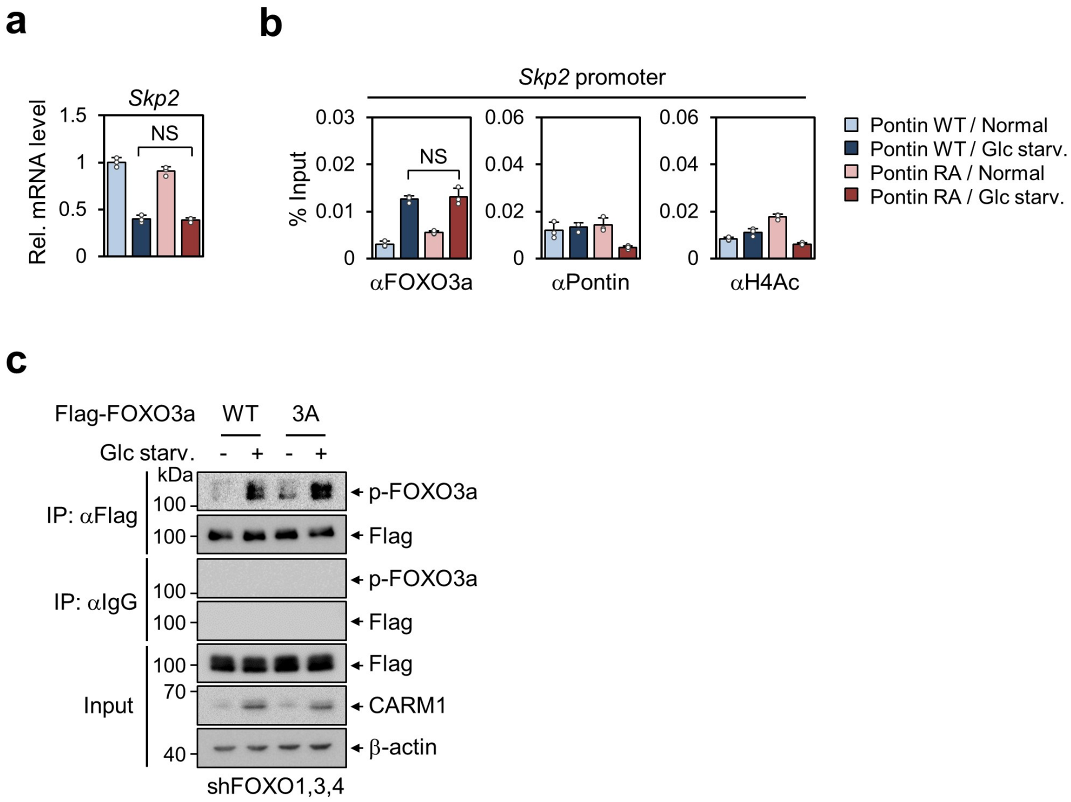


**Supplementary Figure 9. Pontin methylation is not involved in regulation of Skp2 by FOXO3a, related to Figure 8.**

(**a**) Quantitative RT-PCR analysis of *Skp2*. Bars, mean ± s.e.m.; n=3; NS = Non-Significant. Statistics by two-tailed t-test.
(**b**) ChIP assays were performed using anti-FOXO3a, anti-Pontin, and anti-H4Ac antibodies on *Skp2* promoter in Pontin WT or RA-expressing MEFs in the absence or presence of glucose starvation. Bars, mean ± s.e.m; n=3; NS = Non-Significant. Statistics by two-tailed *t*-test.
(**c**) Immunoprecipitation assay between CARM1 and FOXO3a WT or 3A mutant following glucose starvation.
Source data are provided as a Source Data file.

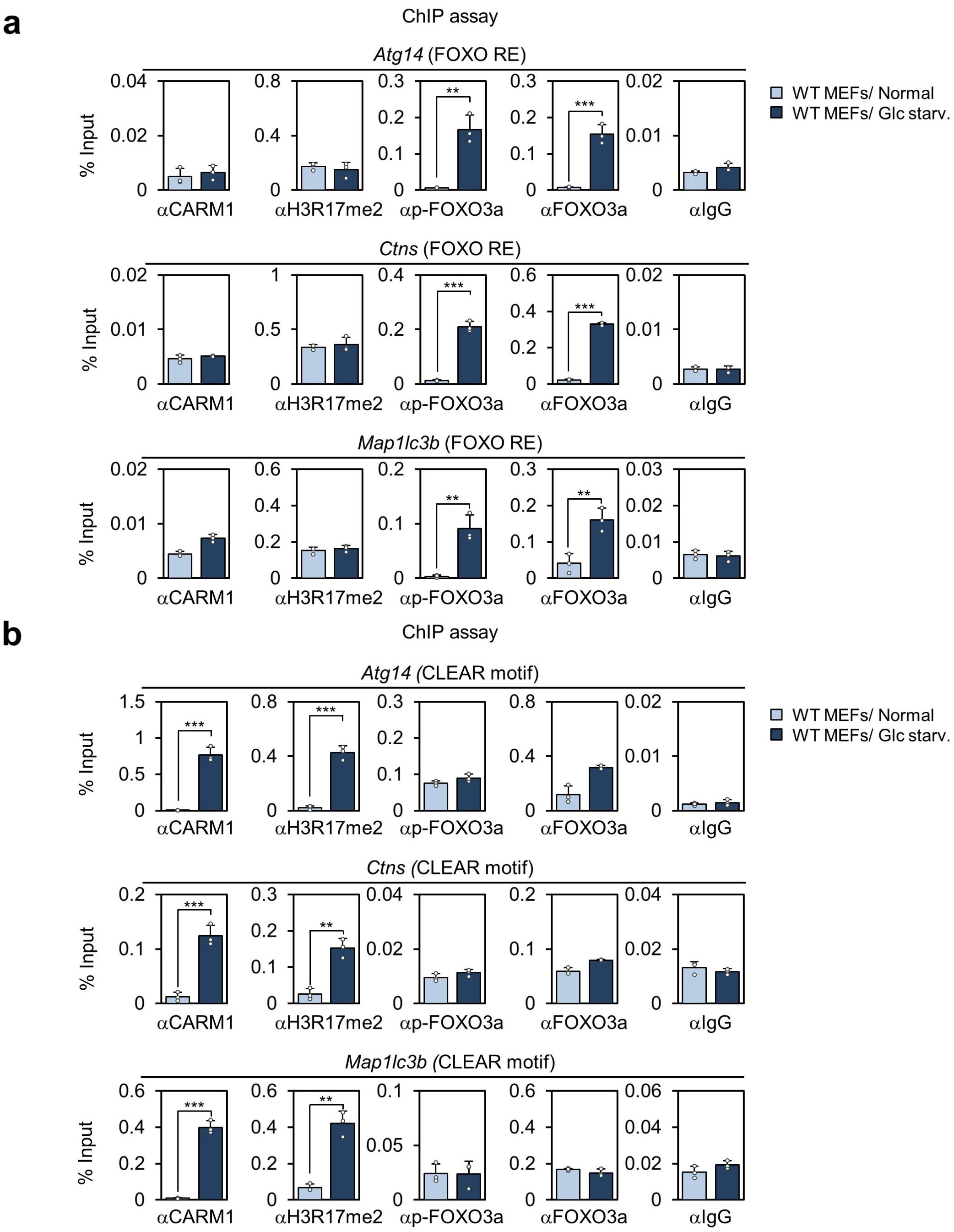


**Supplementary Figure 10. CARM1 is recruited on CELAR motif, but not on the FOXO3 response, related to Figure 8.**

(**a-b**) ChIP assays were performed using anti-CARM1, anti-H3R17me2, anti-p-FOXO3a, anti-FOXO3a, and anti-IgG antibodies on FOXO REs (**a**) or CLEAR motifs (**b**) of *Atg14*, *Ctns*, or *Map1lc3b* genes in WT MEFs in the absence or presence of glucose starvation. Bars, mean ± s.e.m; n=3; ** $p<0.01$, *** $p<0.001$. Statistics by two-tailed t-test.

**Supplementary Table 1. List of protein and peptide sequences identified by LC-MS/MS analysis, related to Figure 1.**

| Accession | MW [kDa] | calc. pI | Score | Description |
|---|---|---|---|---|
| gi530414438 | 63.4 | 6.57 | 428.39 | PREDICTED: histone-arginine methyltransferase CARM1 isoform X1 [Homo sapiens] |
| gi733606247 | 46.0 | 5.12 | 198.96 | tubulin alpha-1C chain isoform b [Homo sapiens] |
| gi197692395 | 50.2 | 6.42 | 118.87 | RuvB-like 1, Pontin, partial [Homo sapiens] |
| gi40041430 | 8.8 | 4.70 | 108.64 | unnamed protein product, partial [Homo sapiens] |
| gi158259801 | 89.8 | 7.33 | 77.28 | unnamed protein product [Homo sapiens] |

| Sequence | Charge | m/z [Da] | MH+ [Da] |
|---|---|---|---|
| ALESSIAPIVIFASNR | 2 | 844.47272 | 1687.93816 |
| ALESSIAPIVIFASNR | 2 | 844.47272 | 1687.93816 |
| ALESSIAPIVIFASNR | 3 | 563.31744 | 1687.93778 |
| ALESSIAPIVIFASNR | 3 | 563.31744 | 1687.93778 |
| ALESSIAPIVIFASNR | 2 | 844.47333 | 1687.93938 |
| ALESSIAPIVIFASNR | 2 | 844.47333 | 1687.93938 |
| AVLLAGPPGTGK | 2 | 540.82434 | 1080.64141 |
| AVLLAGPPGTGK | 2 | 540.82434 | 1080.64141 |
| GLGLDESGLAK | 2 | 530.28772 | 1059.56816 |
| GLGLDESGLAK | 2 | 530.28772 | 1059.56816 |
| GTEDITSPHGIPLDLLDR | 3 | 650.33710 | 1948.99674 |
| GTEDITSPHGIPLDLLDR | 3 | 650.33710 | 1948.99674 |
| LDPSIFESLQK | 2 | 638.84271 | 1276.67815 |
| LDPSIFESLQK | 2 | 638.84271 | 1276.67815 |
| QAASGLVGQENAR | 2 | 650.83350 | 1300.65972 |
| QAASGLVGQENAR | 2 | 650.83350 | 1300.65972 |
| TALALAIAQELGSK | 2 | 693.40356 | 1385.79985 |
| TALALAIAQELGSK | 2 | 693.40356 | 1385.79985 |
| TALALAIAQELGSK | 3 | 462.60510 | 1385.80075 |
| TALALAIAQELGSK | 3 | 462.60510 | 1385.80075 |
| TISHVIIGLK | 2 | 540.84265 | 1080.67803 |
| VEAGDVIYIEANSGAVK | 2 | 867.94867 | 1734.89006 |
| VEAGDVIYIEANSGAVK | 2 | 867.94867 | 1734.89006 |
| VEAGDVIYIEANSGAVKR | 3 | 631.00238 | 1890.99259 |
| YSVQLLTPANLLAK | 2 | 765.94794 | 1530.88860 |
| YSVQLLTPANLLAK | 2 | 765.94794 | 1530.88860 |
| YSVQLLTPANLLAK | 2 | 765.94806 | 1530.88884 |
| YSVQLLTPANLLAK | 2 | 765.94806 | 1530.88884 |
| YSVQLLTPANLLAK | 2 | 765.94800 | 1530.88872 |
| YSVQLLTPANLLAK | 2 | 765.94800 | 1530.88872 |
| YSVQLLTPANLLAK | 2 | 765.95087 | 1530.89446 |
| YSVQLLTPANLLAK | 2 | 765.94830 | 1530.88933 |

**Supplementary Table 2. List of genes from GO term analysis, related to Figure 3.**

| GO Term | Genes |
|---|---|
| GO:0010506 (GOBP) Regulation of autophagy | *KAT8, RAB3GAP2, CLN3, WASH1, ATG14, UVRAG, BNIP3, PINK1, CTSA, RRAGD, FLCN, SIRT1, RRAGC, MAP1LC3B, ULK1, PIKFYVE, TICAM1, TRP53INP1, RAB20, SLC17A9, PIP4K2C* |
| mmu04142 (KEGG) Lysosome | *GNPTG, LAPTM4B, CLN3, ABCB9, AP1G2, CTSA, DNASE2A, ATP6V0B, SLC17A5, NAGPA, TPP1, MCOLN1, ATP6V0D1, CTNS, GGA2, CLN5, CTSF* |
| GO:0005776 (GOCC) Autophagosome | *CLN3, WASH1, MAP1LC3B, ULK1, ATG14, NBR1, TICAM1, TRP53INP1, VPS11, VPS33A, PIP4K2C* |
| GO:0005764 (GOCC) Lysosome | *SLC36A1, UVRAG, CTSA, RRAGD, SLC26A11, VPS33A, RRAGC, TPP1, APOE, MT1, MCOLN1, SLC35F6, VPS11, DPP7, PQLC2, CLN3, ABCB9, MARCH8, DNASE2A, TMEM150A, SLC17A5, UNC13D, NBR1, ARL8A, CTNS, CLN5, CTSF* |
