# Supplementary material for: Pontin arginine methylation by CARM1 is crucial for epigenetic regulation of autophagy
Source: Nat Commun. 2020 Dec 8;11:6297. doi: 10.1038/s41467-020-20080-9 (PMC7722926; doi:10.1038/s41467-020-20080-9)

Figure 1a

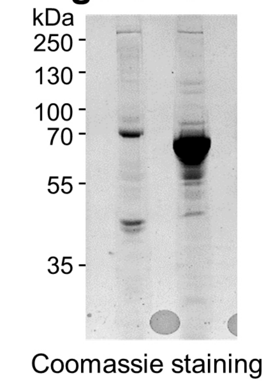

Figure 1b

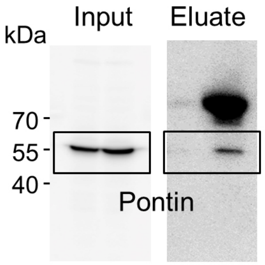

Figure 1c

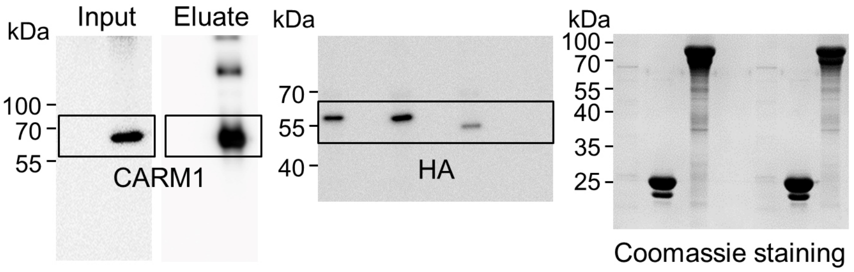

Figure 1d

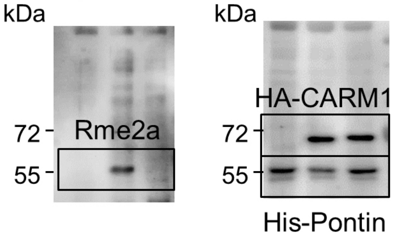

Figure 1e

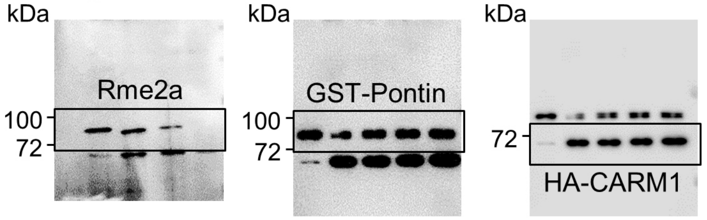

Figure 1f

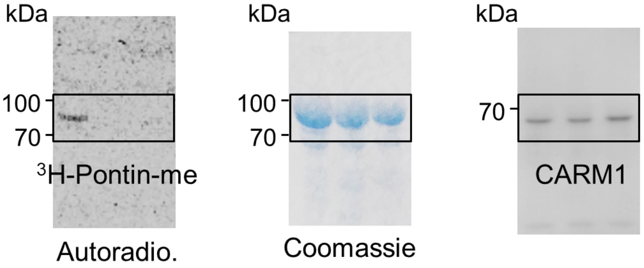

Figure 1i

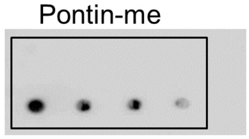

Figure 1j

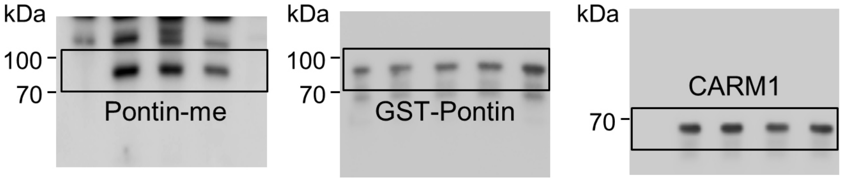

Figure 1k

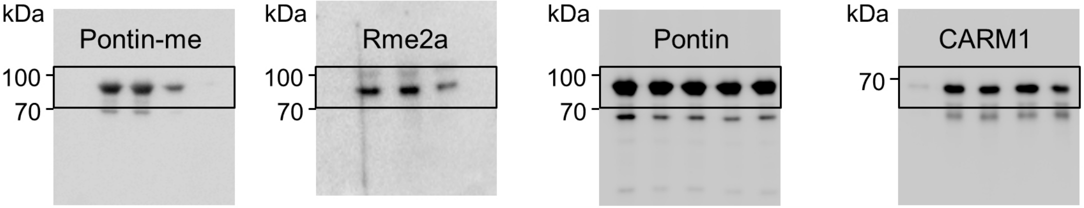

Figure 1l

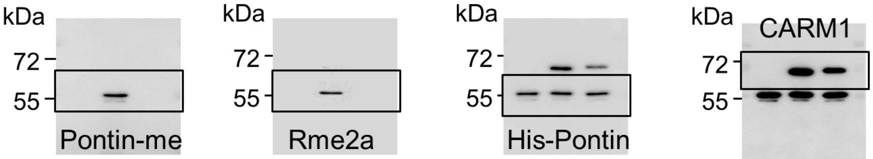

Figure 2a

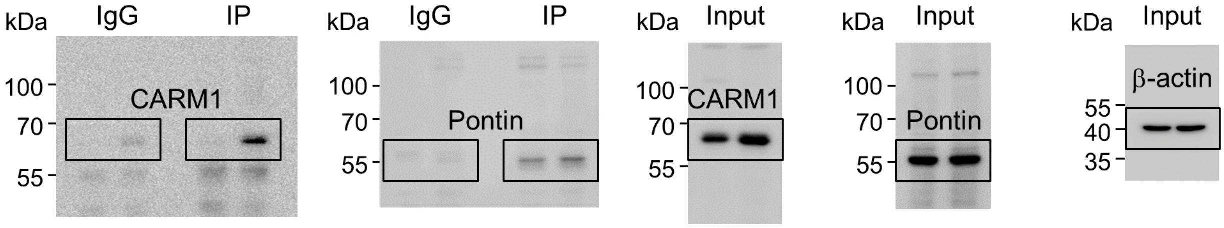

Figure 2b

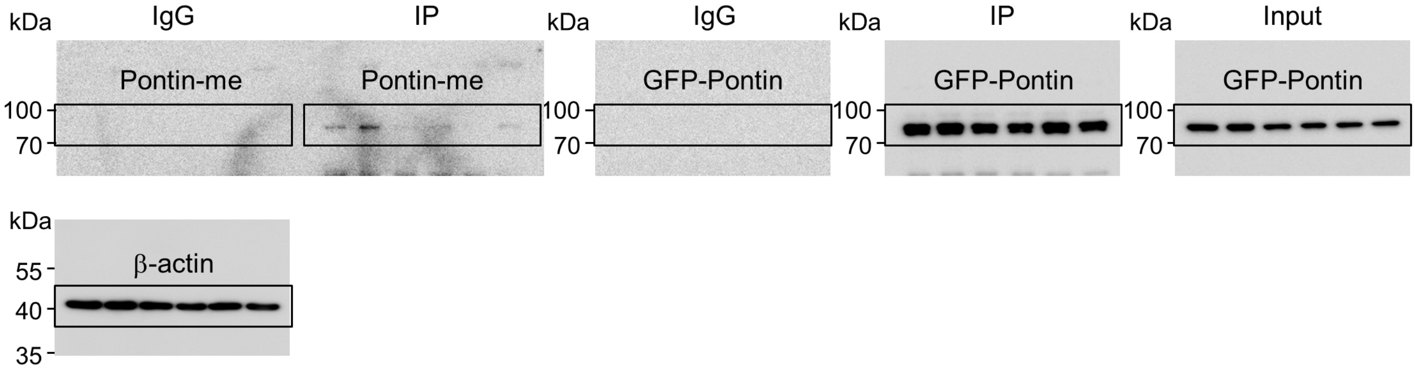

Figure 2c

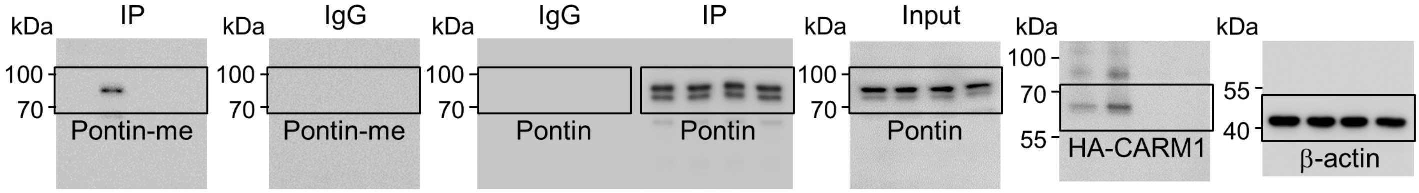

Figure 2d

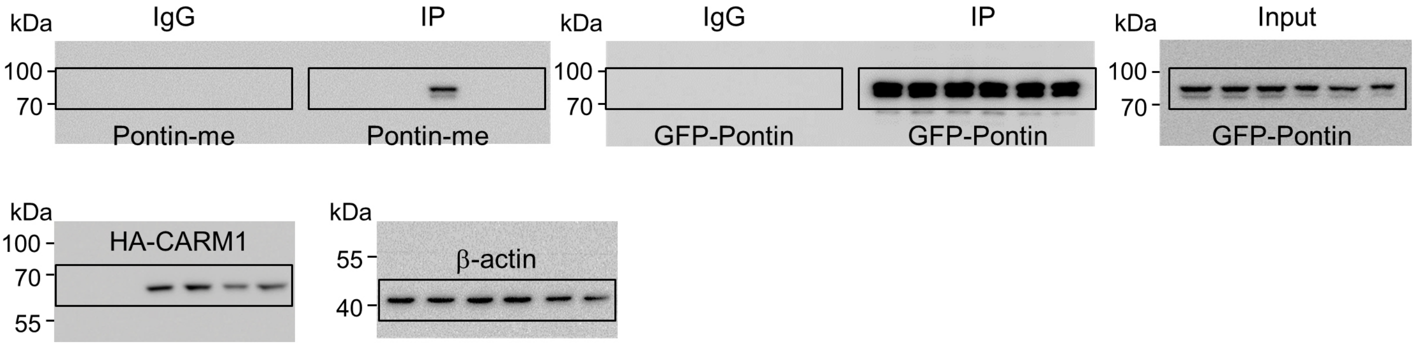

Figure 2e

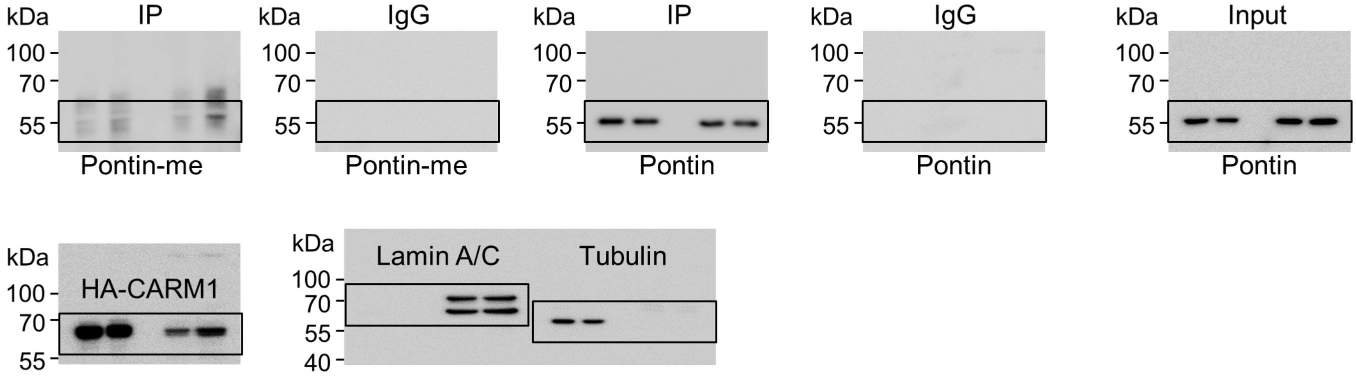

Figure 3b

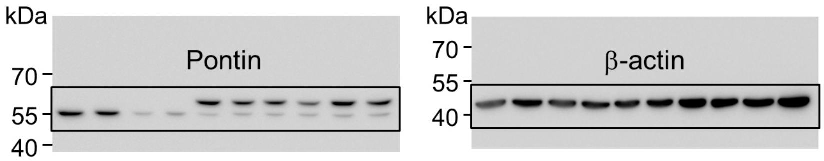

Figure 3c

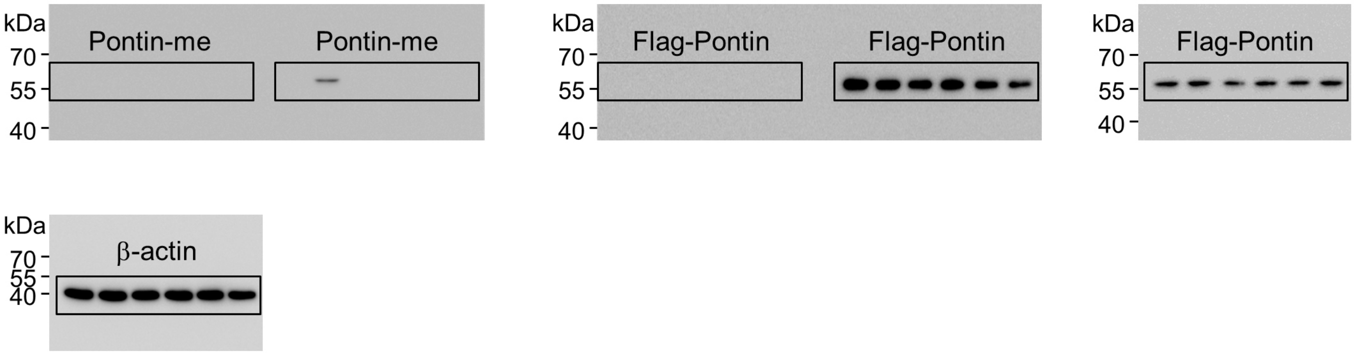

Figure 3e

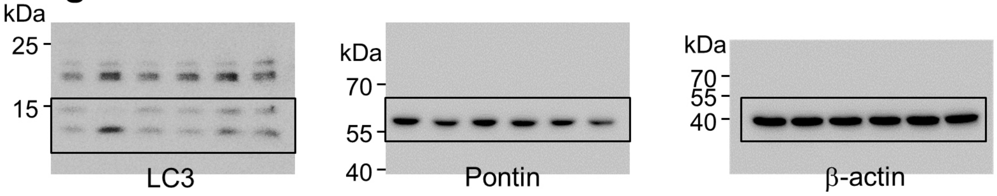

Figure 3g

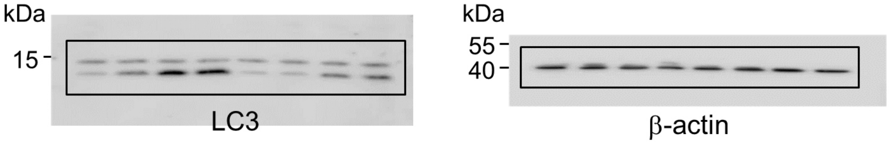

Figure 4g

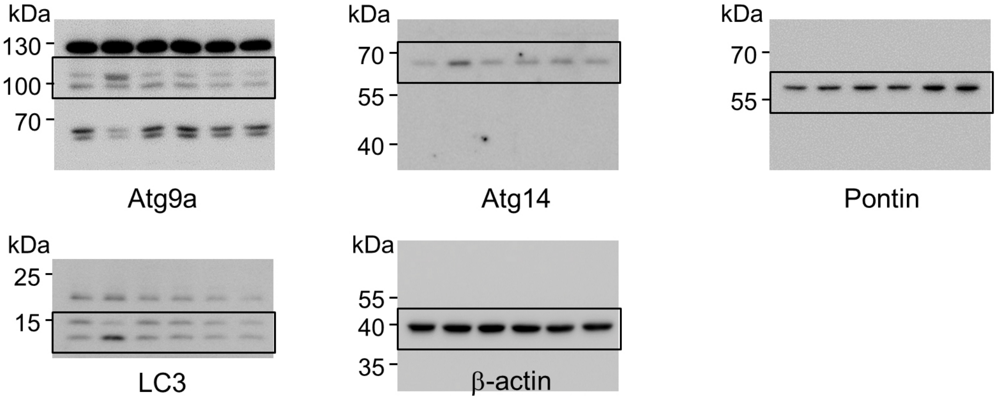

Figure 5c

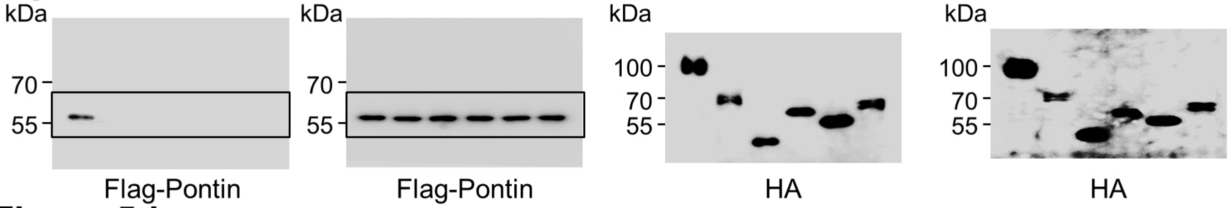

Figure 5d

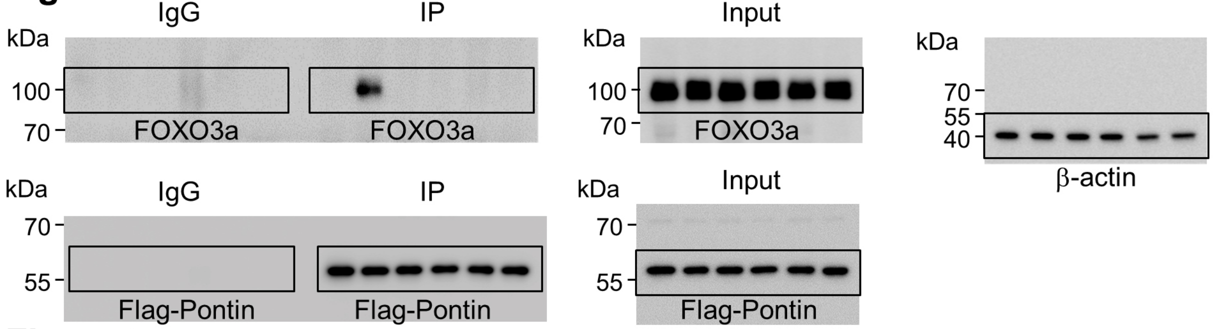

Figure 5e

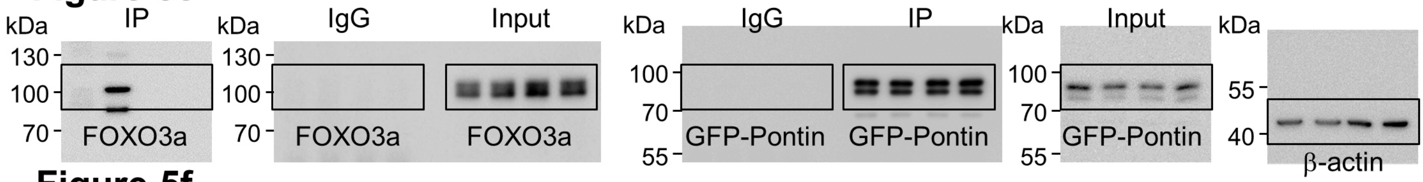

Figure 5f

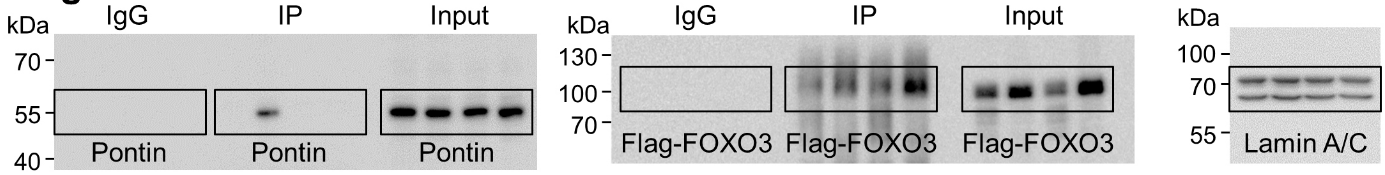

Figure 5g

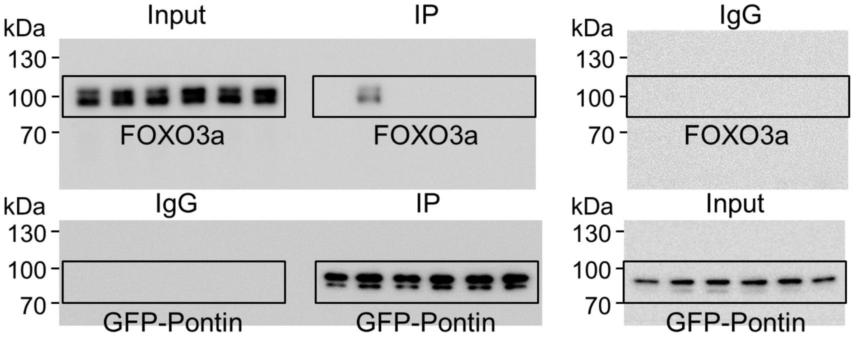

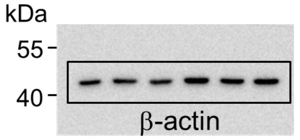

Figure 5i

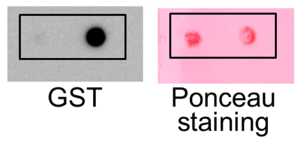

Figure 5h

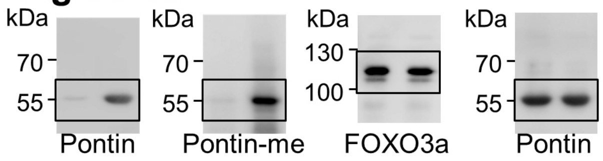

Figure 5k

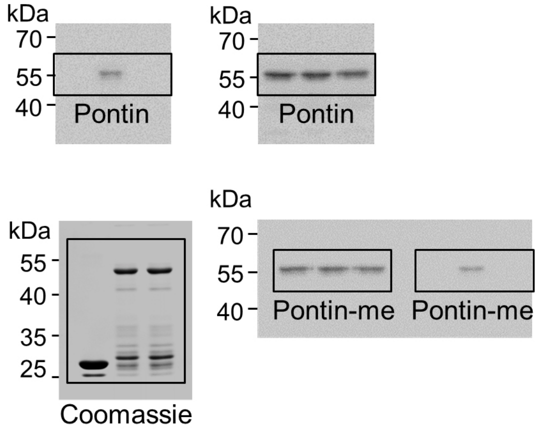

Figure 5j

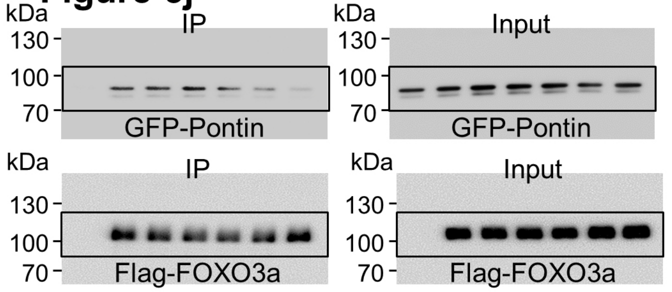

Figure 6f

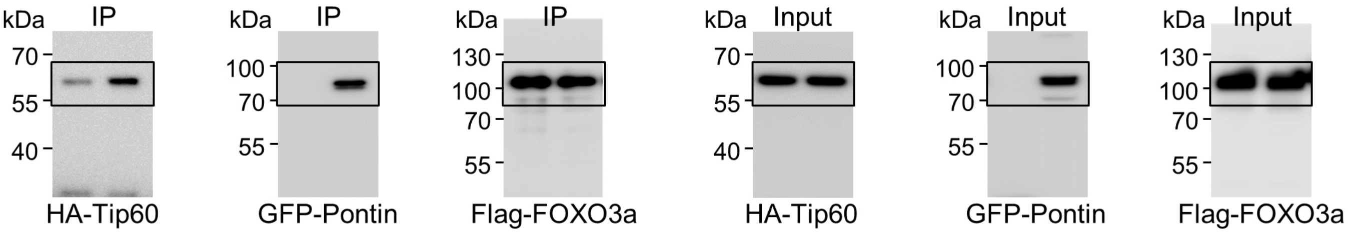

Figure 6g

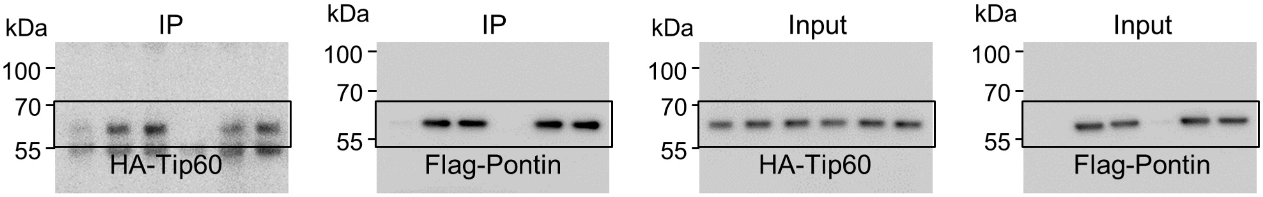

Figure 7a

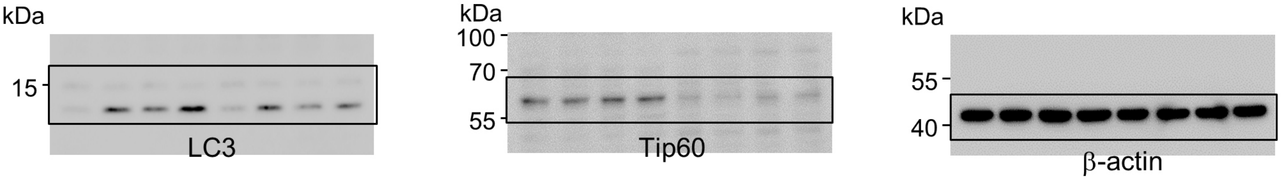

Figure 7e

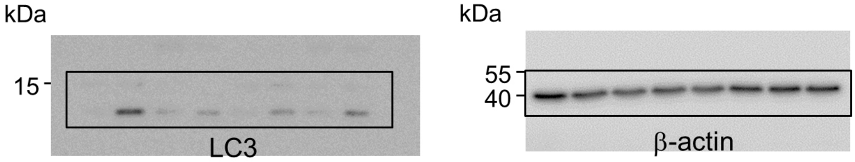

Figure 8c

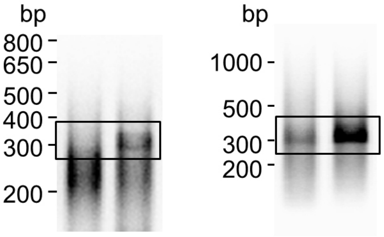

Supplementary Figure 1a

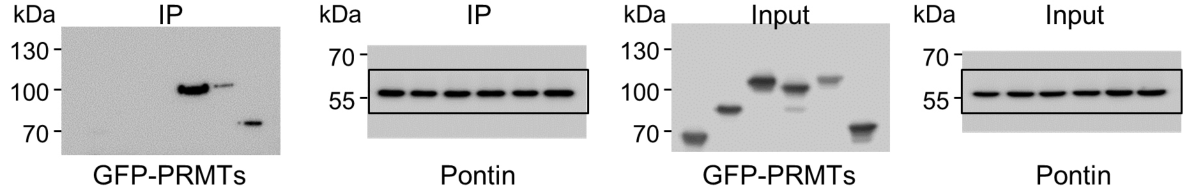

Supplementary Figure 1b

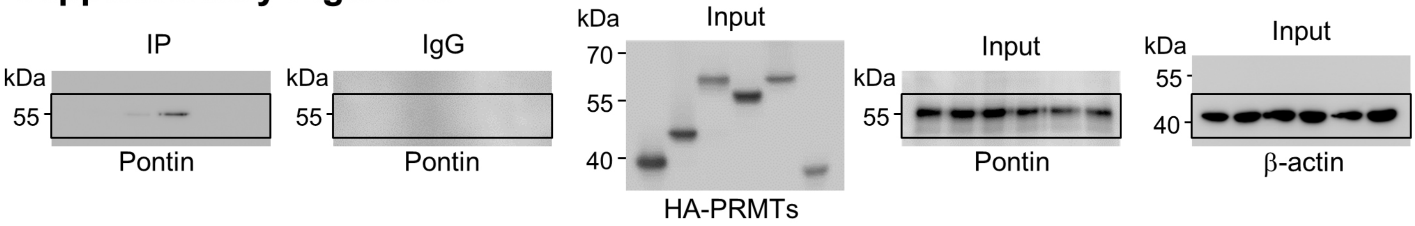

Supplementary Figure 1c

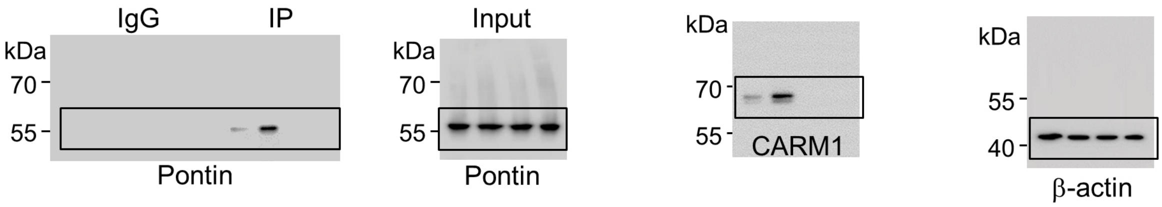

Supplementary Figure 1d

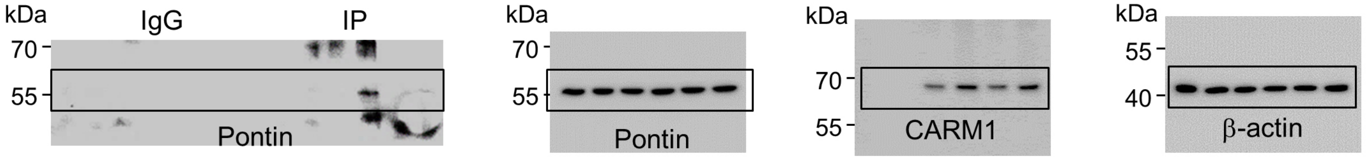

Supplementary Figure 1e

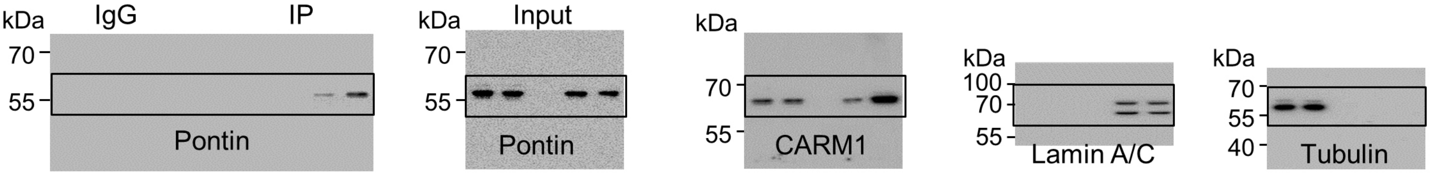

Supplementary Figure 1f

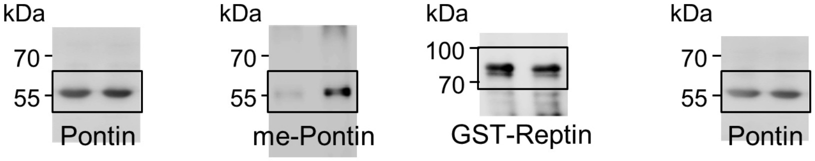

Supplementary Figure 1g

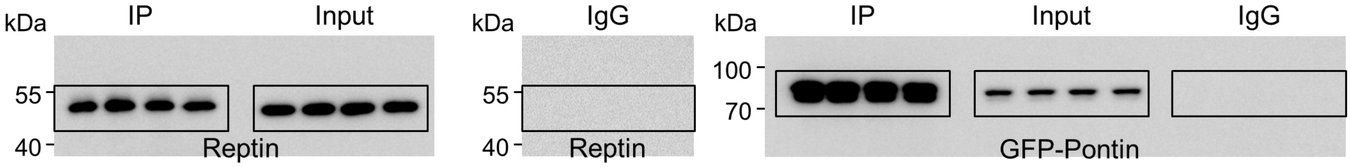

Supplementary Figure 2a

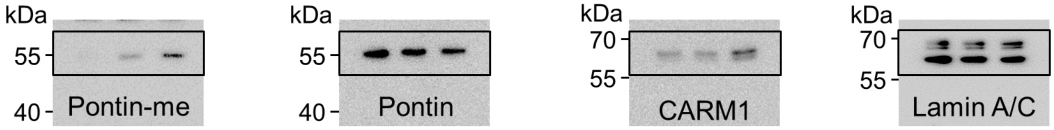

Supplementary Figure 2b

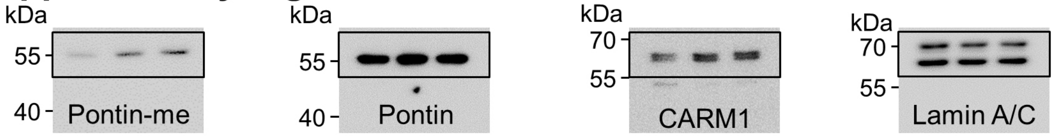

Supplementary Figure 2f

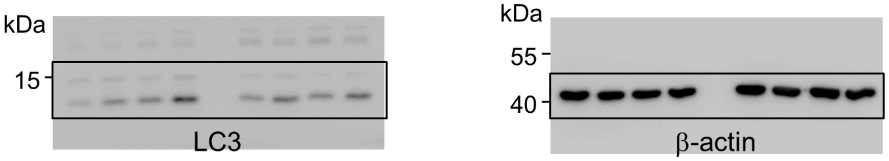

Supplementary Figure 2g

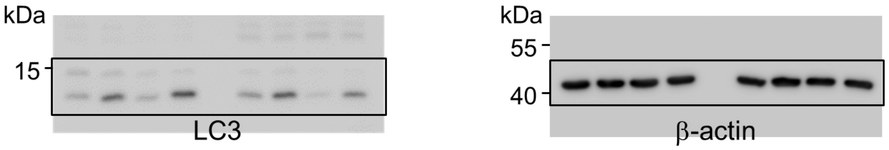

Supplementary Figure 3a

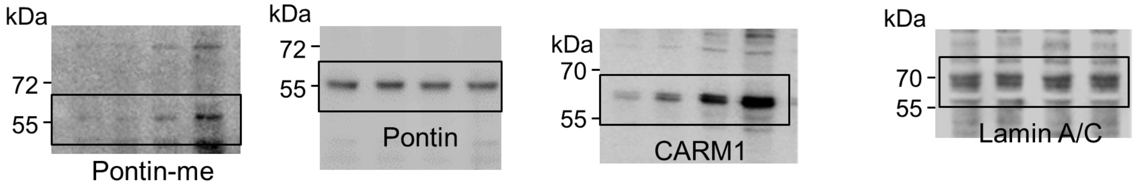

Supplementary Figure 3b

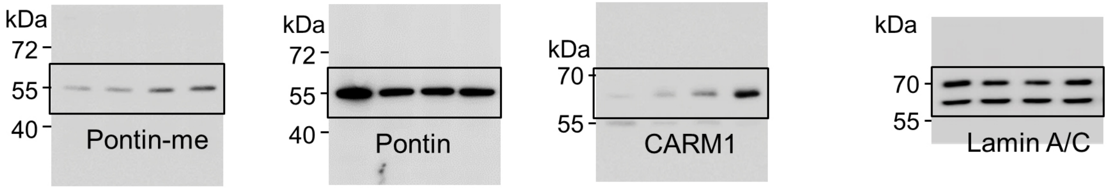

Supplementary Figure 3c

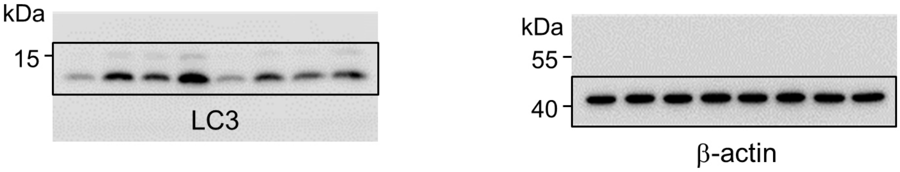

Supplementary Figure 3d

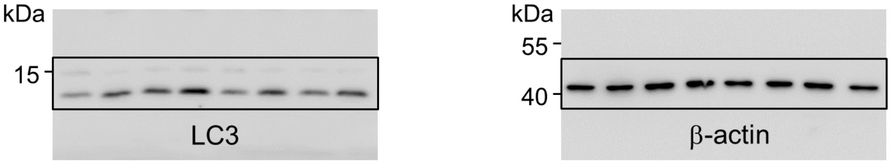

Supplementary Figure 7a

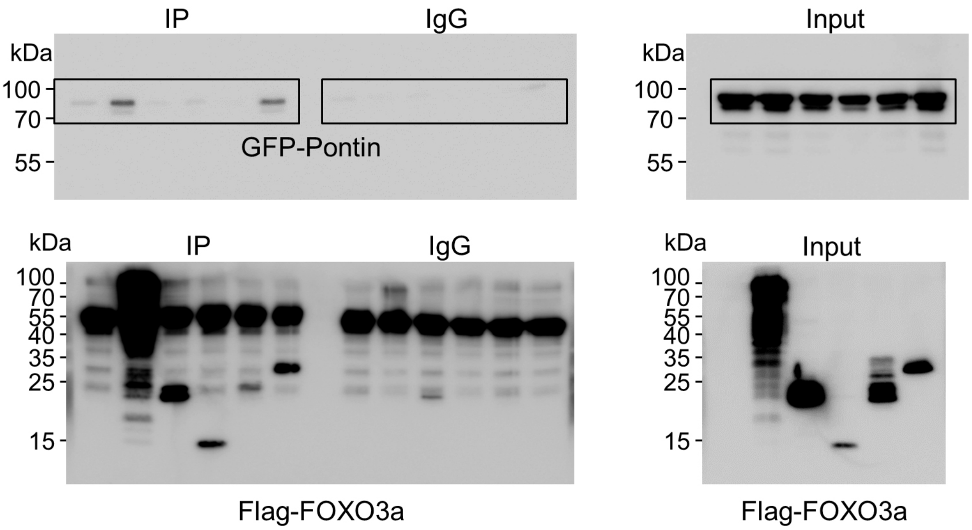

Supplementary Figure 7b

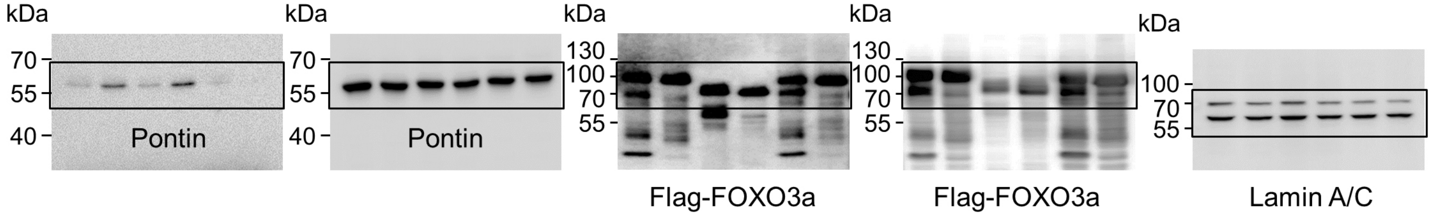

Supplementary Figure 7d

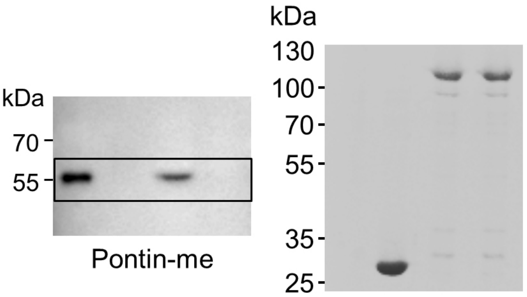

Supplementary Figure 7e

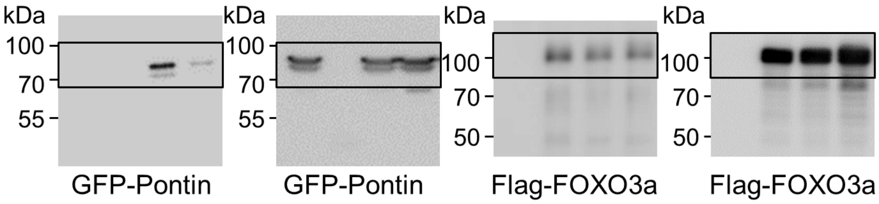

Supplementary Figure 9c

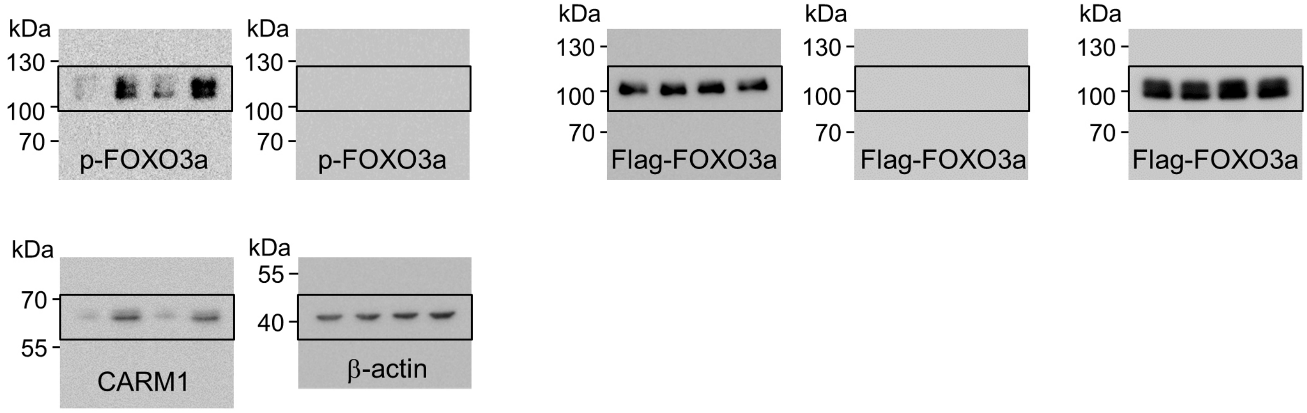

Supplement: Supplementary file 3 — Source Data [file 41467_2020_20080_MOESM3_ESM.zip › Source Data(10_28_20)_YS.pdf]
